# The development of models to predict melting and pyrolysis point data associated with several hundred thousand compounds mined from PATENTS

**DOI:** 10.1186/s13321-016-0113-y

**Published:** 2016-01-22

**Authors:** Igor V. Tetko, Daniel M. Lowe, Antony J. Williams

**Affiliations:** Institute of Structural Biology, Helmholtz Zentrum München für Gesundheit und Umwelt (HMGU), Ingolstädter Landstraße 1, b. 60w, 85764 Neuherberg, Germany; BigChem GmbH, 85764 Neuherberg, Germany; NextMove Software Limited, Innovation Centre (Unit 23), Cambridge Science Park, Cambridge, CB4 0EY UK; ChemConnector Inc., 904 Tamaras Circle, Wake Forest, NC 27587 USA

## Abstract

**Background:**

Melting point (MP) is an important property in regards to the solubility of chemical compounds. Its prediction from chemical structure remains a highly challenging task for quantitative structure–activity relationship studies. Success in this area of research critically depends on the availability of high quality MP data as well as accurate chemical structure representations in order to develop models. Currently, available datasets for MP predictions have been limited to around 50k molecules while lots more data are routinely generated following the synthesis of novel materials. Significant amounts of MP data are freely available within the patent literature and, if it were available in the appropriate form, could potentially be used to develop predictive models.

**Results:**

We have developed a pipeline for the automated extraction and annotation of chemical data from published PATENTS. Almost 300,000 data points have been collected and used to develop models to predict melting and pyrolysis (decomposition) points using tools available on the OCHEM modeling platform (http://ochem.eu). A number of technical challenges were simultaneously solved to develop models based on these data. These included the handing of sparse data matrices with >200,000,000,000 entries and parallel calculations using 32 × 6 cores per task using 13 descriptor sets totaling more than 700,000 descriptors. We showed that models developed using data collected from PATENTS had similar or better prediction accuracy compared to the highly curated data used in previous publications. The separation of data for chemicals that decomposed rather than melting, from compounds that did undergo a normal melting transition, was performed and models for both pyrolysis and MPs were developed. The accuracy of the consensus MP models for molecules from the drug-like region of chemical space was similar to their estimated experimental accuracy, 32 °C. Last but not least, important structural features related to the pyrolysis of chemicals were identified, and a model to predict whether a compound will decompose instead of melting was developed.

**Conclusions:**

We have shown that automated tools for the analysis of chemical information have reached a mature stage allowing for the extraction and collection of high quality data to enable the development of structure–activity relationship models. The developed models and data are publicly available at http://ochem.eu/article/99826.

**Electronic supplementary material:**

The online version of this article (doi:10.1186/s13321-016-0113-y) contains supplementary material, which is available to authorized users.

## Background

The prediction of physicochemical properties is important in the pharmaceutical industry for structure design and for the purpose of optimizing ADME properties. Physicochemical parameters such as logP, pKa, logD, aqueous solubility and many others impact not only drug-related properties but also environmental chemicals such as surfactants, wetting agents and so on [1, 2]. The modeling of these properties is best facilitated by obtaining large, structurally diverse, high-quality datasets. The aggregation and curation of such datasets can be very exacting in terms of extraction of the data from the literature. Redrawing of chemical compounds can be difficult and in many cases they are not available as structure depictions but only in the form of chemical names. Validating the measured property in any meaningful way is difficult but manual inspection can highlight obvious errors with the parameters as captured (vide infra).

Text-mining for the identification and extraction of properties may offer an opportunity to assemble rather large databases of properties harvested from the appropriate corpora. One of the authors (D.L.) has extensive experience with the extraction of chemistry-related information from PATENTS and previous investigations have examined the extraction of chemical reactions [3]. Initial investigations of chemical property measurements contained within the USPTO patent collection indicated the presence of a large number (>100,000) of melting points (MPs), typically within semi-structured experimental sections.

The theme of this memorial issue is focused on the contributions of Jean-Claude Bradley to Open Science and Dr. Bradley had a particular interest in the quality of MP data and he invested significant efforts in investigating this property. His interests were in regards to the value of MP to help in predicting temperature-dependent solubility for solvent selection [4] as well as assembling measured experimental properties as part of an Open Notebook Challenge [5]. He was particularly interested in the quality of experimental MPs reported in the literature and those reported by chemical vendors [6]. He had also worked tirelessly to make a large data collection of over 20,000 MPs available as Open Data [7]. In collaboration with Dr. Andrew Lang, one of the editors for this memorial issue, he made available MP web services [8] providing access to open models for prediction [9] and, prior to his passing, published an open dataset of 28,645 measurements for the community to use to develop models [10].

The prediction of MP remains an important task for cheminformatics studies for a number of reasons [2, 11–17]. It specifically has relevance in the prediction of toxicity but has been observed to correlate with other physical properties such as boiling point, vapor pressure and water solubility [1, 18]. As a result the MP has been used as a descriptor in some of the estimation methods used to predict these properties [1, 19] and therefore the use of reliable MP data, or accurate estimates obtained from high-performing models, can improve the accuracy from such methods. With this in mind we decided to investigate the data mining of property data from an openly available patent corpus, with a focus on the extraction, curation and modeling of MP data.

## Datasets utilized in this work

### Data extracted by mining patent literature

**Fig. 1:**
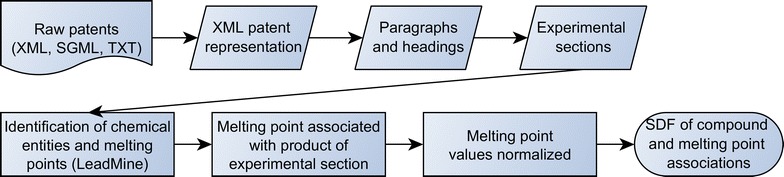
Workflow for extraction of melting point data

The workflow for extracting compound/MP associations is summarized in Fig. 1. All United States Patent and Trademark Office (USPTO) PATENTS available as structured text were downloaded from ReedTech [20] for the period 1976–2014. Patent grants were available for the entirety of this period, while patent applications were available only from 2001 onwards. Complicating data extraction, the format used by the USPTO has varied over time with four significantly different formats being employed (one textual, one SGML and two XML formats). To simplify further handling, the textual and SGML formats were converted to an equivalent XML representation using a LeadMine [21] library function. From these heterogeneous XML representations, headings and paragraphs were extracted from the description section of each patent. The paragraphs are associated with the paragraph number noted in the XML, hence simplifying relating extracted data back to its locations in the original patent. From this point the workflow is the same for all formats of patent. The headings and paragraphs were grouped into experimental sections using the methodology described by Lowe [3]. LeadMine was then used to identify chemical entities and MPs.

**Fig. 2:**
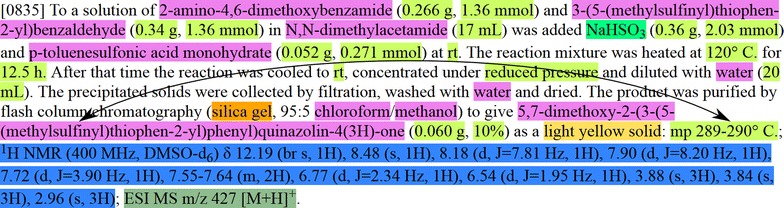
Example of typical experimental section with entities machine-annotated. The entities to associate are shown above

The association of MPs that are in close proximity to a chemical entity (e.g. in a bracket after the chemical), was achieved using a customized version of ChemicalTagger [22]. This customization consisted of adding support for tokens containing spaces (such that a MP measurement could be treated as a single token) and the integration of LeadMine to identify chemical entities and MPs. ChemicalTagger associates properties with chemical entities using a grammar that describes the syntax of a chemical entity with associated chemical properties. In many experimental sections the association of the MP with the synthesized compound is only implicit from the context, i.e. the MP appears at the end of the experimental section along with any other characterization data. In these cases the assumption is made that the MP applies to the compound being synthesized in that paragraph (Fig. 2).

### Melting point recognition

Melting points are efficiently identified by LeadMine using a finite state machine compiled from a formal grammar. The same grammar is also used to generate a parser for identifying the different parts of a MP declaration. The grammar can be summarized as:

FromLiterature? MeltingPoint Qualifier? (Value|Range|MeasurementError) OutcomeQualifier?

Where:

**Table:** 

Term	Examples of text matched
FromLiterature	“lit.”
MeltingPoint	“mpt”, “melting point”, “m.p.”
Qualifier	“>”; “approximately”
Value	“75 °C”, “200 °F”, “one hundred degrees Celsius”
Range	“184–186”, “191.5–192.4 °C”
MeasurementError	“50 ± 1 °C”
OutcomeQualifier	“decomp.”, “with decomposition”, “subl.”

As the grammar accepts numbers both as numerals and decimals, and qualifiers both as symbols and words, the different lexical ways of representing a MP are collapsed into a normalized form that is used for further processing. Values expressed as measurement errors were converted to ranges and all temperatures were converted to degrees Celsius. The original text was retained for reference.

### Extracted data

**Fig. 3:**
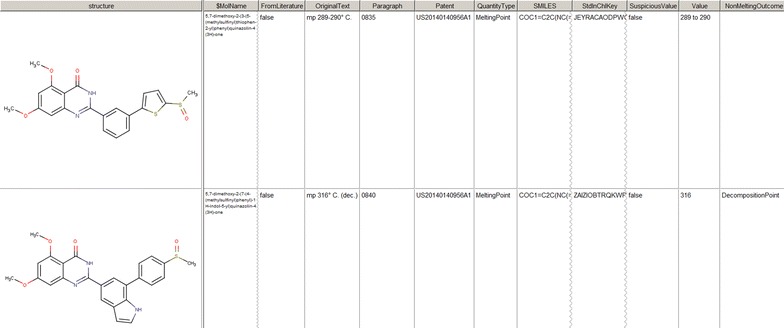
Example of two entries from the resultant SDF

The associations between molecules and melting/decomposition/sublimation points were serialized to SDF format [23] (Fig. 3).

### Suspicious value detection

Melting points that could be automatically detected as being likely to be incorrect were flagged in the SDF. This flag was set for cases where:

- Value was >500 °C;
- Value was a range wider than 50 °C;
- Value was a range where the second temperature was lower than the first temperature.

These heuristics aimed to detect cases where the patent text was likely to be in error e.g. typo, missing decimal point, missing hyphen etc.

### Data filtering

In total 498,985 associations were found in patent grants and 172,886 associations were found in the patent applications. 1498 and 426 associations, respectively, were excluded from the two sets by checking for the aforementioned suspicious value flag. Additionally all compounds that were mixtures i.e. contained more than one connected component, were excluded.

A large number of MP measurements were duplicated across different PATENTS. To avoid duplicates we eliminated records with ΔT ≤ 1 °C differences in reported MP values, which were considered as full duplicates. This procedure eliminated N = 366,532 associations. All other values were considered as multiple measurement values for the same molecule. For each molecule we selected one record, which had MP near to the median experimental value for it. This allowed us to preserve the link to the originating patent, which could then be revisited in case of a problem with each particular record. We also excluded all molecules, which failed with the descriptor calculation programs. The final number of records is shown in Table 1.

**Table 1:** The number of compounds and average properties of molecules of the analyzed datasets and their drug-like subsets

Dataset	Type	Whole set				Drug-like set, % of the total set
		N	Average			
			T (°C)	MW	NA	
PATENTS	Training	241,958	159	357	25	89
Decomposing	Training	13,785	209	358	25	76
Non-decomposing	Training	228,173	155	357	25	93
Bergström	Validation	277	151	295	20.8	92
Bradley	Validation	2878	59	174	11.4	53
OCHEM	Validation	21,832	117	249	16.7	73
Enamine	Validation	22,449	143	223	14.9	91
COMBINED	Validation, merge of four sets	47,436	126	233	15.6	81

*MW* molecular weight, *NA* number of non-hydrogen atoms

### Experimental accuracy of data

**Fig. 4:**
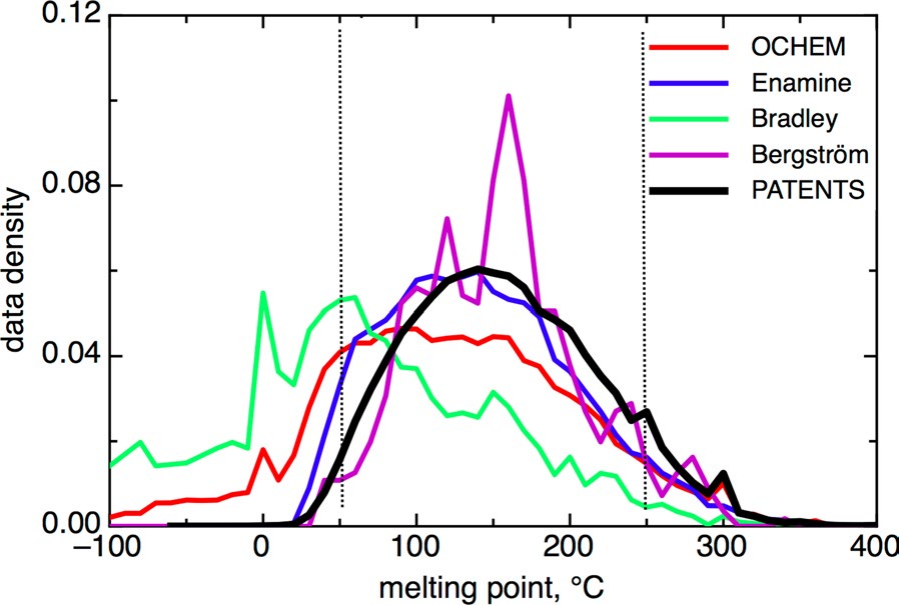
Data distribution in the analyzed sets. The *dashed lines* indicate a defined drug-like region, which covers the MP of >90 % of drugs (Bergström) and chemical provider (Enamine) set as well as 87 % of the compounds from the PATENTS set

The duplicated measurements N = 18,058 were used to estimate the experimental accuracy of MP measurements, which was estimated to be σ = 38 °C. Considering that the procedure to eliminate duplicated records eliminated also molecules having ΔT ≤ 1 °C measured in different experiments, we corrected the observed distribution of values for ΔT = 0 and 1 by using the same number of counts as observed for ΔT = [2, 3] °C interval. This procedure provided σ = 35 °C, which can be used as an average estimation of the experimental accuracy of MP measurements across multiple experiments. This value incorporated the uncertainties due to polymorphism of chemical compounds, uncertainty and difficulties with experimental measurements as well as possible text-mining errors. For example, the distribution of MP values from PATENTS literature had peaks at 250 and 350 °C thus indicating that measurements were either stopped at these temperatures and threshold values were reported or simply that at these temperatures an estimated value within a fairly broad range was entered (i.e. an accurate MP was not required per se, see Fig. 4). All of these uncertainties decreased the accuracy of MP measurements.

**Fig. 5:**
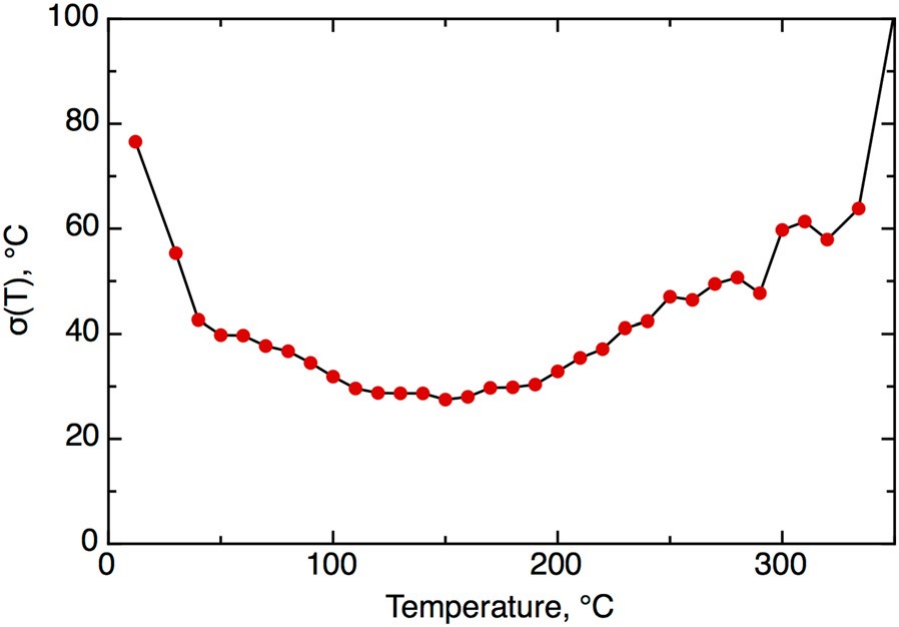
The experimental accuracy of the data as a function of the MP temperature. Each point averaged at least 50 measurements. The graph was built using N = 18,058 differences in the MP temperatures and was rescaled to match the average experimental accuracy of σ = 35 °C. Compounds with MP <0 °C, most of which were data processing errors, were excluded

It is interesting that the experimental accuracy depended on the MP value. A binned plot of the accuracy as a function of the MP temperature indicates that measurements with higher and lower temperatures were less reproducible (Fig. 5). The measurements in the drug-like region of [50, 250] °C were estimated to have an experimental measurement error of σ = 32 °C.

### Validation datasets

Four other MP data sets were used to validate the models developed in this work. These datasets were taken from our previously published study [11]. The “Bergström” dataset contained drug-like molecules [17]. The “Bradley” dataset [24] contains doubly curated data collected by Open Notebook science community members. The OCHEM and Enamine datasets [11] comprised MP values collected from datasets available via the Online Chemical Modeling Environment (http://ochem.eu) and provided by Enamine Ltd. These datasets did not have any common overlapping compounds. The compounds overlapping with any of these four sets were removed from the PATENTS set. We also used a combined dataset (COMBINED) composed of the OCHEM, Enamine, Bradley and Bergström sets to simplify analysis of performances for several studies.

### Drug-like subsets

In our previous study we showed that compounds with MP in the range 50–250 °C contributed the majority of compounds in drug-like collections [11]. Table 1 and Fig. 4 confirm this observation and indicate that about 90 % of compounds from the PATENTS, Enamine and Bergström data sets are covered by this temperature interval. Indeed, it is unlikely to find in a drugs dataset compounds with MPs below room temperature (i.e. liquids) or with very high MPs, e.g. >500 °C. The former may have low affinity and specificity while the latter are likely to be non-soluble. Therefore, the pharma industry is mainly working with compounds from the “drug-like” region of chemical space and the accuracy of prediction for compounds from this region is the most important for drug discovery.

The statistics of all datasets is provided in Table 1. There is a correlation between the average molecular weight (MW) and average MP of compounds. This result is in agreement with the known problem of decreasing solubility of compounds in drug discovery for large molecules. The compounds with MP from the PATENTS dataset contributed molecules with the largest MW and thus MP. The compounds from the Bergström dataset had the second largest MPs. The Bradley dataset, which was composed of many general chemical industry compounds, had the smallest average MW and MP values.

## Methods

The consensus modeling approach, which was also applied in our previous studies [11, 25, 26], was used to develop models. The descriptors were calculated using 13 descriptor packages, which cover different representations of chemical structures from simple fingerprints and a count of chemical groups, to packages offering a wide variety of descriptors types, such as Dragon [27] and Adriana [28]. All of these descriptor types are implemented within the OCHEM platform [29]. Below we briefly overview the used descriptors (see also Table 2). The detailed information about each set of descriptors can be found on the OCHEM [30].

**Table 2:** Analyzed sets of descriptors

Package name	Type of descriptors^a^	Number of descriptors	Matrix size, billions	Number of descriptors after filtering	Non-zero values, millions	Sparseness^b^
EFG	Binary	595	0.18	347	3.1	33
QNPR	Integer	1502	0.45	1040	6.3	49
MolPrint	Binary	688,634	205	197,367	8.1	7200
E-state count	Float	631	0.19	487	10	14
Inductive	Float	54	0.02	39	11	1
ECFP4	Binary	1024	0.31	1021	12	25
ISIDA	Integer	5886	1.75	2275	18	37
ChemAxon	Float	498	0.15	114	23	1.5
GSFrag	Integer	1138	0.34	469	24	5.7
CDK	Float	239	0.07	182	27	2
Adriana	Float	200	0.06	139	32	1.3
Mera, Mersy	Float	571	0.17	235	61	1.1
Dragon	Float	1647	0.49	911	183	1.5

^a^The dominating type of descriptors within the corresponding package

^b^Average number of zero entries per one non-zero value of the descriptor matrix

E-state [31] refers to electro-topological state indices that are based on chemical graph theory. E-state indices are 2D descriptors that combine the electronic character and topological environment of each skeletal atom and bond. The environment of atoms and bonds determine their type. In this study after a preliminary analysis we found that E-state indices and just counts of atom and bond types defining E-state indices produced similar results. Since development of models with E-state counts was faster, the counts were used.

### In silico design and data analysis (ISIDA) fragments

These 2D descriptors are calculated with the help of the ISIDA fragmenter tool [32]. Compounds are split into substructural molecular fragments (SMF) of (in our case) lengths 2–4. Each fragment type comprises a descriptor, with the number of occurrences of the fragment type as the respective descriptor value. In this study, we used the sequence fragments composed of atoms and bonds.

### GSFragments

GSFrag and GSFrag-L [33] are used to calculate 2D descriptors representing fragments of length ${\text{k}} = 2 \ldots 10$ or ${\text{k}} = 2 \ldots 7,$ respectively. Similar to ISIDA, descriptor values are the occurrences of specific fragments. GSFrag-L is an extension of GSFrag: it considers labeled vertices in order to take heteroatoms of otherwise identical fragments into account.

### CDK v. 1.4.11 (3D)

The Chemistry Development Kit (CDK) [34] is an open source Java library for structural chemo- and bio-informatics. It provides the descriptor engine, which calculates 246 descriptors containing topological, geometric, electronic, molecular, and constitutional descriptors.

### Dragon v. 5.5 (3D)

Dragon is a software package from Talete [27] that calculates 3190 molecular descriptors. They cover 0D–3D space and are subdivided into 29 different logical blocks. Detailed information on the descriptors can be found on the Talete website (http://www.talete.mi.it/).

### ChemAxon (v. 5.10.4) descriptors (3D)

The ChemAxon [35] Calculator Plugin produces a variety of properties. The properties encoded by numerical or Boolean values were used as descriptors [29]. They were subdivided into seven groups, ranging from 0D to 3D: elemental analysis, charge, geometry, partitioning, protonation, isomers, and others.

Adriana.Code v.2.2.6 [28] (3D), developed by Molecular Networks GmbH, calculates a variety of physicochemical properties of a molecule. The 211 resulting descriptors range from 0D descriptors (such as MW, or atom numbers) to 1D, 2D, and various 3D descriptors.

Mera/Mersy (3D) developed by chemosophia [36] included geometrical, energy characteristics, molecular symmetry and chirality and physicochemical descriptors [37].

### QNPR descriptors

Quantitative Name Property Relationship (QNPR) are 1D descriptors, which are directly based on IUPAC names or SMILES text string representation of molecules. The descriptors are calculated by splitting the respective string of all possible continuous substrings of a fixed length. In our study the substrings of length one to three characters calculated by splitting SMILES structures were used. The minimum frequency of an occurrence of each substring within the dataset was five.

ToxAlert [38] extended functional groups (EFG) [39] included 583 groups covering different functional features of molecules. The groups are based on classifications provided by the CheckMol software [40], which was extended to cover new groups, in particular heterocycles [39].

ECFP4 descriptor circular fingerprints [41] were calculated using ChemAxon software v. 5.10.4. These descriptors are widely used as part of the Pipeline Pilot software [42].

MolPrint descriptors [43] are circular fingerprints which employ Sybyl MOL2 atom types. They are based on counts of MOL2 atom types around each heavy atom of the molecule and enumerate all atom environments present in a molecule.

#### Machine learning methods

In our previous work [11] we found that ASNN [44] and SVM [45] methods provided significantly higher accuracy of MP predictions compared to other tested methods while the accuracy of models developed with both methods was similar.

The same two approaches were initially used in this study. However, the training of large datasets requires significant computational resources and can take a long time. The LibSVM supports parallelization, which can be easily enabled by editing a few lines of code and linking the code with appropriate libraries. This feature was used for LibSVM and all calculations were performed on servers with up to 32 cores simultaneously. Considering that all models were validated using a fivefold cross validation approach, we were using up to 6 × 32 = 192 cores per one task simultaneously thus allowing fast processing of the data. The implementation of ASNN did not offer this feature. Therefore, after initial analysis LibSVM was used to develop all models using radial basis function (RBF) kernel. The most recent version LibSVM v. 3.20 was used [46].

#### Optimization of LibSVM parameters

The application of the SVM method required an optimization of three parameters, C, γ and ε. The LibSVM manual proposes to use a grid search based on an internal cross-validation (CV) procedure to optimize them. This grid optimization procedure is implemented as part of OCHEM. The full run includes 1693 individual LibSVM calculations using different combinations of three analyzed parameters. This step requires a significant computational time. Moreover, it is also parameterized: the user should indicate which fraction of data should be used for the optimization to speed up the search. When using 1 % of a randomly selected training data set we found that, surprisingly, the same parameters (C = 64, γ = 1, ε = 0.00391) were optimal for 10 out of 13 descriptor sets. However, parameters selected with such a small data subset could be suboptimal for the whole dataset. Considering that the selection of optimal parameters for this dataset practically did not depend on the used descriptors, we decided to perform the optimization using 50 % of the training set for only one descriptors set. The EFG were selected as the set having the smallest number of non-zero values (Table 2). The optimization required about 15,000 core-hours (>600 days of calculations on a single-core computer) and identified another set of parameters (C = 256, γ = 1, ε = 16), which was used for the final analysis. This second set of parameters provided on average smaller training and validation set errors and calculated models with the smaller number of support vectors. For example, models based on Dragon descriptors were 316 and 219 Mb (in a zipped file format) when developed with the first and the second set of SVM parameters, respectively.

#### Unsupervised descriptors selection

Before the development of models, descriptors, which had two or fewer non-zero values for the whole training set were eliminated. Moreover, descriptors which were inter-correlated with a linear correlation coefficient of R^2^ > 0.95 were grouped together and only one descriptor from the group was selected for model development. This unsupervised filtering does not use any information about the target property and thus does not introduce selection bias [47], which could provide chance correlations.

#### Validation of models

The models developed using the PATENTS dataset were validated using fivefold CV as described in details elsewhere [48]. In this approach each model is built using 4/5 of the compounds from the initial training set. The remaining 20 % of compounds are predicted and are used to estimate the accuracy of the models. By repeating the model building five times one can calculate predictions for all molecules from the initial dataset. These predictions are used to estimate the CV accuracy of the model. The final model is built using all training set data.

For classification of molecules that melt and decompose we used bagging validation [49]. Since the number of decomposing molecules was ca. 6 % of the dataset and thus much smaller compared to non-decomposing ones, a specific implementation, so called stratified bagging learning, was selected [50]. It is one of the most successful methods to work with an imbalanced dataset. In the stratified bagging approach the molecules of the smallest class are selected using sampling with replacement to form a set of the same size as the class is. The same procedure is also used for the larger class but the number of selected samples is limited to that of the smaller class. The resulting training set used is thus double the size of the number of samples in the smaller class. The selection of samples is repeated for each developed model used in the bagging protocol. The predictions are calculated for samples which were not included in the respective training sets and are averaged over all calculated models. The bagging models were developed using N = 64 models.

#### Consensus modeling

Consensus modeling was shown to be an essential approach to calculate high prediction accuracy for the previous study [11]. A simple average of models

1 $$\bar{y} = \frac{1}{n}\sum y_{i}$$

where *n* is the total number of models and *y*_*i*_ is an individual prediction was used to develop the consensus model in that study. In this study the individual models were developed each with 1 of 13 sets of descriptors, which are described above. This approach contributed highly predictive models, as reported in the previous studies [11, 25, 26, 51–53], including Rank-I submission models [52, 53] for the ToxCast challenges organized by EPA and NIH.

In this study two other additional methods were also analyzed. The first approach was averaging by model accuracy

2 $$\bar{y} = \sum \frac{{w_{i} y_{i} }}{{\sqrt {\sum (w_{i} )^{2} } }};\quad w_{i} = 1/RMSE$$

where RMSE was the root mean squared error of the model. In the second approach a consensus model was developed using the predictions of individual models as descriptors for a multiple linear regression model (MLRA).

### Handling of intervals and ranges

A majority of MP values were reported as intervals or ranges. We used the average or threshold value for the development of the LibSVM models.

### Reproducibility of models

The OCHEM web site was developed with the idea of delivering full reproducibility of modeling efforts. Thus, each model has details of the configuration which was used to create it. The configuration includes options for data standardization, descriptor calculation and pre-processing as well as the parameters for the configuration of the machine learning methods, e.g. LibSVM in this study. The configuration can be exported in an XML human-readable format using the “Export configuration XML” link available on the model profile. If a user wishes to exactly reproduce the model the exported configuration can be uploaded to the model development web page (OCHEM menu: “Models”/“Create a model”) using “Import an XML model template” or just use the configuration of the previous model (“Use another model as a template”). Once one of these options is used the model can be submitted to perform calculations without a need to specify any other parameters and will use exactly the same workflow as the original model. The only exception is the Consensus model which will require repeating the steps used for the model development manually (the options from XML configuration will be automatically pre-set), due to the technical differences in the implementation of this model. It should be noted that the calculation of large models requires significant CPU resources. Users are therefore allowed to submit tasks with a maximum number of molecules which is proportional to the number of bonus points they have collected (i.e. during the process of registering, uploading data, developing and publishing models and participating in data moderation). The limitation on the number of molecules per task is also useful to prevent possible challenges from inexperienced users who can initiate very large calculations by mistake. As an example, a non-registered and validated registered user can submit models with up to 1000 and 10,000 molecules per task, respectively. It is always possible to contact the web administrator (first author of the manuscript) to increase this limit for some specific projects. A detailed protocol used for the development of the consensus MP model for the PATENTS dataset is provided as Additional file 1.

### Automatic filtering of outliers

OCHEM provides tools for the automated recognition and filtering of errors. It assumes that the distribution of errors, i.e. differences between predicted and experimental values, is governed by a Gaussian distribution N(0, σ) with a dispersion which equals the σ = RMSE. Molecules with large errors between the predicted and calculated MP values are unlikely to be produced with a Gaussian distribution and are considered to be outliers. The probability of finding a molecule with an error between the predicted and measured values of larger than two σ is p < 0.05. For the dataset with N = 229k molecules one can expect to have 22.9 ≈ 23 molecules for p = 0.0001. For the model with RMSE = 40 °C this value corresponds to errors which are larger than about 3.8σ and thus 150 °C between predicted and calculated values. If instead of N = 23 we detect e.g. l N = 163 molecules with such large errors, we can assume that the vast majority of outliers are either experimental errors or there are some problems with the data or with the model itself. If the outliers are indeed errors then their exclusion can improve the quality of the models. Of course, by removing the outliers we will also remove a number of “good” data points (in this case N = 23), which could have large errors due to the statistical properties of the dataset. Contrary to the removal of outlying data, the removal of “good” molecules will decrease the data set size and thus will decrease the quality of the model. The ratio of identified outliers to that expected by chance corresponds to the signal-to-noise ratio (SNR). For the considered example the SNR is 163/23 ≈ 7 i.e. out of seven molecules identified for this p-value, only one can be explained by statistical properties of the data. Thus, for this SNR the removal of seven outlying points will also remove one “good” data point.

### SetCompare

This utility uses a hyper-geometric distribution to identify the probability that observed the ratios of a particular feature (e.g. alert) in two analyzed sets could happen by chance [25].

## Results

The modeling of large datasets represents many challenges with respect to the required computational time, storage of descriptors and the calculated model as well as the selection of appropriate machine learning algorithms, which can handle such data. The descriptor packages analyzed in this study calculated different numbers of descriptors (see Table 2). The largest matrix was contributed by MolPrint descriptors. It had an initial size of 688, 634 × 197, 367–200 × 10^9^ (0.2 trillion points) which decreased to 60 billion after the unsupervised filtering. The training of a model with hundred thousand descriptors is infeasible with computational algorithms, which operate with the full matrix. Examples of such algorithms include neural networks, multiple linear regression analysis and partial least squares.

However, the matrix produced by MolPrint descriptors is a very sparse one: only one out of more than 7000 descriptor entries was non-zero (Table 2). This matrix had the third smallest number of non-zero descriptors after the EFG and QNPR. Such sparse data can be analyzed using kernel-based methods. These approaches deal with the pairwise similarity of molecules and thus can efficiently work with sparse data by performing calculations using non-zero entries only. The support of a sparse data format is efficiently realized in LibSVM making this method easily applicable to this type of data. OCHEM software also supports a sparse data format thus making it possible to fully utilize the power of the LibSVM method.

The EFG, despite their high dimensionality, had only 3.1 million non-zero values, and provided the fastest calculations. The calculation of one model for these descriptors (without optimization of LibSVM parameters) required about 120 core-hours. The Dragon descriptors contained the largest number >183 million non-zero values, and required the longest calculation time of more than 1000 core hours.

### Comparison of the accuracy of models developed using PATENTS dataset with previous models

As in our previous study [11] the model developed using E-state indices calculated the lowest RMSE for the training set and provided one of the best results for the four validation sets (see Additional file 2: Table S1, Table 3). The largest errors of models were calculated using ECFP4, MolPrint and Inductive descriptors, which had a cross-validation RMSE of >50 °C for the training set.

**Table 3:** RMSE of models for prediction of different sets

Method	PATENTS set	Bergström	Bradley	OCHEM	Enamine	COMBINED
PATENTS E-state	38.3 ± 0.1 (36.1)^a^	34 ± 1 (31)	62 ± 1 (33.7)	48.5 ± 0.4 (36.2)	40.8 ± 0.3 (35.2)	45.9 (35.6)
PATENTS Consensus all ten models	37.8 ± 0.1 (34.1)	34 ± 1 (31)	78 ± 1 (32.2)	54.2 ± 0.4 (34.1)	40.4 ± 0.3 (33.9)	50.5 (33.9)
PATENTS Consensus five best models	37.0 ± 0.1 (33.7)	33 ± 1 (31)	71 ± 1 (31.3)	50.1 ± 0.4 (33.8)	39.4 ± 0.3 (33.5)	46.9 (33.6)
OCHEM consensus	–	34 ± 1 (31)	33.9 ± 0.6 (33.1)	–	40.1 ± 0.3 (34.6)	–
Enamine consensus	–	36 ± 2 (33)	73 ± 1 (33.9)	51.9 ± 0.4 (36.6)	–	–

^a^Values in parentheses are calculated for compounds with experimental MP values in [50; 250]  °C “drug like” interval. They had the same or lower confidence intervals, which are not indicated

A consensus model was built as a simple average of all models with an exception of the three aforementioned models, which had CV RMSE >50 °C. It decreased the RMSE for CV and test set predictions in the range of 1–2 °C compared to the results based on E-state descriptors. This model in its design (a simple consensus average of ten individual models) was the best match to the model developed in our previous study thus allowing their straightforward comparison. The new model provided similar or lower errors for the drug-like subsets compared to the consensus models developed with individual OCHEM or Enamine sets. For example, a consensus model developed with the OCHEM dataset predicted drug-like subsets of the Bradley and Enamine set with RMSEs of 33.1 and 34.6 °C, respectively. The model developed with the PATENTS dataset predicted them with RMSEs of 32.2 and 33.9 °C, respectively. For the Enamine dataset the absolute difference in model errors RMSE = 0.7 °C is significant (p < 0.05) due to the large number of molecules in this set.

The accuracy of the consensus model developed using the PATENTS dataset was low for the whole Bradley set despite it having a low RMSE for the drug-like subset of this set. This result was due to the absence of molecules with MP <0 °C in the PATENTS set. Indeed, there were only few molecules with an MP <0 °C in this set and, moreover, most (all) of these molecules were likely to be experimental errors (see below the section regarding the filtering of outliers). Because of the insufficient coverage of this region of values the model was unable to predict molecules with low MP values, which constituted about 25 % molecules of the Bradley set. The low accuracy of the model for Bradley set (RMSE = 78) is in agreement with the similar error (RMSE = 73) of a consensus model based on the Enamine dataset (see Table 3) [11]. The Enamine set also did not have compounds with MP <0 °C and a model based on this set failed to predict the whole Bradley set despite the fact that it had excellent prediction ability for its “drug like” subset (Table 3).

There were about 5 and 6 % molecules with MP >250 °C in the PATENTS and COMBINED sets respectively. The PATENTS consensus model calculated a large CV RMSE = 61 °C and an even higher RMSE = 74 °C for the PATENTS and COMBINED subsets respectively. The prediction of compounds from this temperature range therefore remains a challenging task.

This analysis demonstrates that models developed using text-mined MP data from PATENTS provide an excellent prediction performance, similar or even significantly better than the results based on manually curated data used in previous studies. Since the patent corpus continues to grow quickly we can envisage that if the workflow and data processing pipeline is applied on an ongoing basis then the dataset will continue to grow and it will be at a much faster rate than manual extraction and curation will allow. While this procedure has only been reported for MP extraction and modeling in this work we can imagine utilizing the same procedure for other physicochemical properties such as multi-solvent solubilities, logP and other available parameters. The success for these parameters is yet to be proven.

### Analysis of different methods to perform consensus averaging

A simple consensus averaging is often used by many researchers, including ourselves, to improve the quality of models by agglomerating predictions of individual models [11, 48, 54]. Is it possible to achieve even better results by using more sophisticated averaging methods? We analyzed three approaches described in the methods section by applying them to n-best models, which were ranked in order of decreasing CV RMSE. The accuracy of predictions of the various models was estimated for the drug-like subset of the PATENTS and COMBINED set.

The average of five models based on E-state, Fragmentor, CDK, ChemAxon and QNPR descriptors calculated the lowest RMSE of 33.7 °C for drug-like subsets of the PATENTS and COMBINED datasets and thus provided an improvement of 0.4 °C compared to the results obtained when averaging ten models.

The models calculated using the weighted average had exactly the same performance for all subsets up to the average of five models. Indeed, since the accuracies of individual models were very similar and their weighted combination did not improve results compared to the simple average. For combinations of a large number of models, the weighted average sometimes provided smaller RMSEs of about 0.01 log units, which was not significantly different compared to the simple average.

The application of MLRA regression on the predicted values did not improve this result and the same RMSE was calculated for the drug-like subset of the COMBINED set. Thus, both studied strategies did not provide an improvement compared to the use of a simple arithmetic average of models.

The important result of this analysis was that the averaging of few models with the highest prediction ability could improve results compared to the averaging of all models.

### Analysis of compounds, which decompose during melting

A number of data points (see Table 1) from the PATENTS collection contained annotation about the thermal decomposition (pyrolysis) of chemical structures. The MW and number of non-hydrogen atoms of decomposing structural were practically identical to other molecules. The CV RMSE for a subset of molecules that decomposed was 47.7 °C, i.e. significantly larger compared to the 36.5 °C calculated for the subset of molecules without the decomposition. The median MP for decomposing compounds was 210 °C as compared to 155 °C for the whole dataset (Table 1). Thus, the lower prediction accuracy for these compounds could partially be due to the higher average MP, which is more difficult to predict.

The SetCompare tool identified that molecules containing acids (carboxylic, phosphonic and α-amino acids), primary amines, tetrazoles, and a number of other groups, were overrepresented in the group of compounds, which decomposed with the heating. The identified overrepresented groups are available for review online at http://ochem.eu/article/99826. Phosphonic acids and α-amino acids were among the most overrepresented groups in the set of decomposing compounds. They were present in only 0.4 % compounds (0.1 % phosphonic and 0.3 % α-amino acids) in the whole set but contributed about 4 % of all compounds in the decomposing set. Thus, the presence of one of these groups increased the probability of a compound to decompose by more than ten times. Compounds with a nitroso group were also about 9 times overrepresented in the decomposing set. The propensity of these three groups to decompose is well known. Already Dunn and Brophy [55] studied the decomposition of the amino acids and their contribution to the uncertainty associated with determination of their MPs. The decomposition of phosphonic acid and its esters has been actively studied in toxicological chemistry since it results in the release of highly toxic phosphine, PH_3_ [56]. Compounds with nitroso groups are well known for their ability to decompose with a release of high energy, which makes them very important for the development of explosives (including dynamite).

The propensity of a compound to decompose versus melt is different properties. We therefore expect that better models should be calculated by considering each property independently.

### Modeling to predict compound decomposition

A model to predict the decomposition point of molecules was developed using the same protocol and SVM parameters selected for the whole PATENTS set. As with the analysis of the whole set of compounds the best accuracy of the individual model was calculated using E-state descriptors (RMSE = 43.2 °C). A consensus model based on the average of five models calculated the lowest RMSE = 42.3 °C. This error was lower than the CV RMSE calculated for the molecules within the whole PATENTS set (Table 4). Indeed, this dataset was more internally consistent thus contributing a better prediction ability of the models. However, the higher CV errors calculated for the decomposition point indicated that this property is even more difficult to predict than MP. This model calculated a higher RMSE for prediction of molecules from both the COMBINED and non-decomposing PATENTS set. This result was expected since both properties describe different physical effects.

**Table 4:** RMSE of the consensus models developed with different subsets of the PATENTS set

PATENTS subset used to train model	PATENTS subsets		COMBINED
	Decomposing	Non-decomposing	
Non-decomposing + decomposing	47.7 (40.5)^a^	36.5 (33.4)	46.9 (33.6)
Decomposing	42.3 (38.9)	64.3 (62.9)	94.9 (70.4)
Non-decomposing	51 (43.1)	36.3 (33.3)	46.5 (33.3)

^a^Values in parentheses are calculated for compounds with experimental MP values in the [50; 250] °C “drug like” interval

### Prediction of MP of compounds cleaned from decomposing molecules

The models calculated for the MP set with excluded decomposing molecules calculated a lower CV RMSE and also a lower RMSE for predictions of the COMBINED set molecules. The increase in the accuracy of 0.1–0.3 log units for both sets was not statistically significant. Of course, it calculated higher RMSE for the prediction of decomposing molecules.

These results indicate the separation of molecules into two classes, i.e. those that decompose and those that do not decompose allowed for the development of better predictive models for each property. Unfortunately, such information is generally unknown for new molecules. A classification of compounds into those that decompose and do not decompose during melting could help to identify both classes of compounds. Moreover, such information can also be useful for the handling of chemical compounds.

### Classification model to predict decomposing compounds

A model was developed using the same sets of descriptors for all molecules from the PATENTS database, which were classified on non-decomposing and decomposing classes. We used stratified undersampling bagging [50] since the decomposing molecules corresponded only to 5.5 % and thus the dataset was highly imbalanced. This approach has demonstrated its high prediction power also for analysis of large chemical datasets [25, 56, 57]. Since the training datasets contained just double the number of decomposing molecules the SVM calculations were fast. Three descriptor sets (E-state, CDK and Fragmentor) had balanced accuracy above 75.5 % with the best one, E-state, having 78.1 %. The use of WEKA [58] implementation of decision trees (J48) improved balanced accuracy for Fragmentor descriptors from 75.8 to 77.6 %. A consensus model based on this decision tree and two SVM models achieved an accuracy of 79.6 ± 0.2 % for the whole set. The E-state model or the consensus model can be used to predict the fate of the molecules.

### Detection of outlying molecules

The automated data extraction from PATENTS resulted in a number of systematic errors in the data, which needed to be cleaned and filtered. As mentioned in the methods section a lot of efforts were devoted to cleaning up the data set during extraction from the literature. Data modeling was very useful during this step. Following data upload to OCHEM we performed modeling and reviewed outlier molecules. After finding and correcting a common pattern, which was leading to errors, data extraction was repeated.

For example, many records in PATENTS had a MP reported as “235-2360” (i.e. the decimal point after 236 was missed). This would be filtered out both due to the range being implausibly large and due to one of the values being implausibly high. Other “errors”, which could easily be corrected by a human, e.g. “159-62”, “160-2”, “82-82,5” (i.e. a comma instead of a dot) were addressed by introducing rules to handle these non-standard forms. Actually, the reporting of MP values as intervals, thus having two values instead of a single reported value, was beneficial to find and eliminate errors in the data.

Some of the problems with collected values were difficult to recognize and eliminate. They could originate from rare types of errors and/or simply be misprints. For example, one of the obvious erroneously reported values was “Mp. −383 °C”, which was a misprint of the minus sign. Another case included missing or incorrect decimal points in the MP values, e.g. “Mp. 236” or “Mp. 236” instead of “Mp. 23.6” and “Mp. 236”, respectively, which contributed noise to the MP values in the high or low temperature region.

Table 3 indicates the performances of consensus models and individual sub-models calculated for the different number of excluded outlying molecules as described in the Methods section. The RMSEs for the PATENTS set were reported for the whole set, i.e. also including the outlying molecules, which were excluded for different p values. This was done to have a simpler comparison of results. Thus the results for the PATENTS set COMBINED prediction of molecules from the validation sets and prediction of the outlying molecules using the final models developed with the respective training set. The reported results and tendencies did not change if we used the PATENTS sets with molecules excluded for different p-values.

The filtering of outlying compounds for *p* in the range of 0.001–0.01 improved prediction accuracies of individual models for both the PATENTS and the COMBINED sets (Table 5). The RMSE of most individual models decreased by about 0.1–1 °C log units for both sets. The degree of improvement depended on the descriptors used. Thus, the exclusion of outlying molecules, which distorted the training procedures, contributed models with higher prediction accuracies.

The improvement in model performance for the whole COMBINED set was larger compared to the results calculated for the drug-like subsets. The distribution of the excluded outlying molecules filtered using, e.g. p = 0.001, indicated a bimodal distribution of their MPs with peaks at 60 and 280 °C, i.e. from the regions outside or on the border of the drug-like region. Thus, increasing the quality contributed to the higher prediction accuracy of models for these regions of chemical space.

It is interesting that similar to our previous study [11] the removal of outlying compounds practically did not affect the performance of the consensus models for the drug-like subsets. Thus, a combination of individual models cancelled the biases of individual models introduced by noise in the experimental data. This result confirms that consensus averaging is a powerful method to increase the accuracy of individual models.

The consensus model provided an improvement, ΔRMSE = 1 °C, for the prediction of molecules outside of the drug-like space for the COMBINED set thus confirming the aforementioned conclusions about the influence of the outlier filtering on the data quality for molecules with this range of MP values.

The number of outlying molecules identified for *p* = 0.1 (N = 21,928) was less than expected for this *p* value, N = 22,208 for Gaussian distribution. Thus, the majority of identified data for this threshold suggested that outliers could just appear due to the statistical properties of the data and their removal can lead to deterioration of the model quality. This can be observed by the fact that RMSEs calculated for the “drug-like” and the whole COMBINED set start to increase for this p-value.

**Fig. 6:**
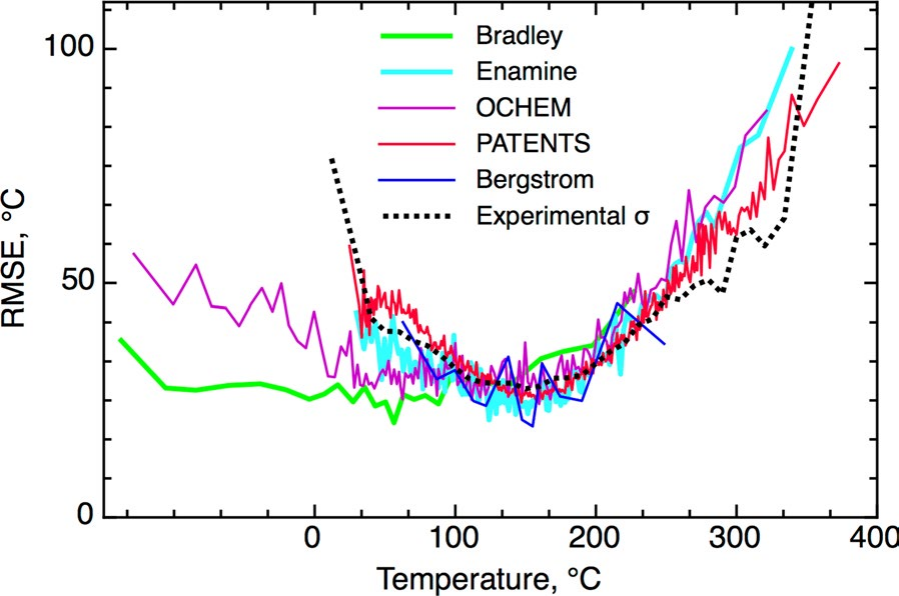
The CV RMSE for different subsets of the final model as a function of MP. Each point on the plot is an average of at least N = 100 predictions with the exception of the Bergström set (N = 20)

The CV RMSE error for the PATENTS set, 36.3 °C, was in good agreement with the estimated experimental accuracy of σ = 35 °C. Moreover, for the drug-like region the estimated σ = 33.3 °C, and calculated errors, CV RMSE = 33 °C, were also very similar. Thus, the developed consensus model achieved the experimental accuracy of the MP data (Fig. 6).

### Analysis of the final model

The final models were developed by pooling data from all five datasets analyzed in this study. The outlying molecules were filtered using p = 0.01. The final consensus model was compared (Table 6) with the model developed using the COMBINED set in our previous publication [29].

The combination of PATENTS and COMBINED sets decreased the RMSE by 0.6 °C for the drug-like subset of the COMBINED set as well as also for the four individual subsets from the previous study. Thus enlargement of the training set increased prediction power of the models according to the CV protocol. The RMSE error calculated for the Bergström set is the lowest published value for this set and it is about 30 % smaller compared to 44.6 °C reported in the original study of Bergström et al. [17].

Figure 6 shows that the CV RMSE of the individual subsets as a function of temperature increases for all sets of high temperatures (MP >250 °C). This decrease in the accuracy of predictions for this region is qualitatively similar for all five analyzed datasets and is in agreement with the decrease of the experimental accuracy of MP data as estimated for the PATENTS set. Thus, the accuracy of prediction of MP for the high temperature region was limited by the accuracy of experimental data.

The experimental accuracy of data was also the limiting factor for the prediction accuracy of the model for PATENTS set for MP <50 °C. The predictions of MP for the Bradley dataset were of higher accuracy in this region. This result can be explained by the different quality of data measurements for this data set. Indeed, Dr. Bradley collected for this dataset only measurements that had highly reproducible published MP values: the values were only kept if there were multiple measurements and the range of values was between 0.01 and 5 °C inclusive. It is also interesting that only a few compounds in this set had MP values of >250 °C, thus indicating the difficulties of identifying reproducible measurements for high MP values.

The developed consensus model estimates both the applicability domain [59] and the accuracy of the prediction for new compounds based on the CONSENSUS-STD distance to model [44, 59]. This distance to model corresponds to the disagreement (standard deviation) of the individual predictions of models in the consensus model [44]. It was found as the most reliable approach to estimate the accuracy of predictions in several benchmarking studies [44, 60, 61].

### Analysis of models based on few descriptors

The MP is used as a parameter for several models, e.g. solubility assessment [19] or as a parameter of multiple solvent models to simulate the accumulation and degradation of chemicals in different solvents, based on a number of explicit mathematical models for the transfer and degradation of molecules [62, 63]. In the absence of the MP values a default value is frequently used, e.g. Syngenta [64] uses MP = 125 °C for their solubility model. In the PATENTS dataset the average MP value was 155 °C, which can be probably used as a better estimation of MP for drug-like compounds. The use of this value as a model prediction for all compounds gave an RMSE = 65.7 °C, which can be used as the null hypothesis for MP prediction. The MW and the number of carbon atoms (NC) had significant linear Pearson correlation coefficients, R = 0.172 and R = 0.136 respectively, relative to MP. The MLRA model developed with both these descriptors MP = 117 + 0.142 × MW − 0.79 × nC achieved an RMSE = 64.7 °C. This model, however, can hardly be considered as an improvement of the null hypothesis model for any practical application.

For another analysis we calculated the Pearson coefficient of correlation between MP and descriptors. The highest negative R = −0.484 and positive correlation R = 0.481 with MP were obtained for the of0ug and ef0ug 3D descriptors [65, 66] calculated using the Mera program [37]. The first descriptor corresponds to the number of electrons participating in the orbital overlap of the carbon atoms. The second one is its complement, which indicates the number of free electrons for the carbon atoms, which do not participate in the overlap. Thus, both of them measure the degree of hybridization of the molecules. The use of the single descriptor of0ug in the model MP = 513–1260*of0ug produced an RMSE of 57 °C. It can be proposed as a single descriptor model for the estimation of MP of compounds.

The nAtomP, which calculates the number of atoms in the largest π-chain, was found as the most highly correlated descriptor, R = 0.371, provided by the CDK package [34]. The second descriptor of the same package, f_MF_, R = 0.357, characterizes the complexity of the molecules. This descriptor is calculated as the fraction of the size of the molecular Bemis and Murcko framework [67] versus the size of the whole molecule and was introduced to predict the promiscuity of chemical compounds [68]. This descriptor is defined in a [0, 1] interval and it is equal to 1 if a molecule does not have side chains.

The two best descriptors calculated by Adriana.CODE [28] 2DACorr_PiEN_3 and 2DACorr_PiEN_4 are 2D π electronegativity-weighted autocorrelation descriptors calculated for topological distances 3 and 4 [69]. Both of these descriptors had R = 0.359.

The number of rings and resonance counts (number of resonance structures of a molecule) were also two highly correlated descriptors (R = 0.355 and R = 0.354) calculated using ChemAxon. Unsaturation and saturation indexes Ui (R = 0.349) and Uc (R = 0.325) were the two most highly correlated molecular property descriptors calculated by the Dragon software. The MP also correlated with more simple descriptors, such as the number of nitrogen atoms (R = 0.322).

The analysis of the most correlated descriptors indicates that many of them are strongly related to the π-system of electrons and thus had the possibility to interact through π-interactions. For example, the presence of side chains decreases f_MF_ and thus the ability to perform such interactions and the formation of crystal structures. Possibly, the same effect contributes to formation of agglomerates in solution thus leading to the promiscuity of chemical substances observed by Yang et al. [68] The same change decreases the number of rings as well as the number of atoms in the largest π-chain (relative to the overall size of the molecule) as well as other electronic parameters of the molecule.

However, the aforementioned effect is not the only one contributing to the MP of compounds. Indeed, we built a linear MLRA using the best 100 and ten descriptors. The models RMSEs 48.1 ± 0.1 (100 descriptors) and 53.6 ± 0.1 (ten descriptors) were more than 10 °C higher compared to those calculated using SVM methods. Thus, while analysis of the individual descriptors is important to understanding the major effects influencing the property, their non-linear interactions, as captured by the machine learning methods, are important to derive the predictive models.

To some extent the comparison of results associated with the MLRA and SVM methods, and the conclusion about the advantage of the latter approach could be biased due to the use of the different number of descriptors used by both models. In order to better evaluate it we developed SVM models using the descriptors selected with MLRA for the PATENTS set for five descriptor sets contributing to the consensus model. The RMSEs of SVM models developed using exactly the same descriptors as those used in MLRA models were on average 7 ± 1 °C lower than the RMSEs of the MLRA models. Thus, the difference in the prediction performances of the SVM and MLRA models was mainly due to the ability of the SVM approach to better handle the non-linearity of data.

### Models and data availability

The final models based on the E-state descriptors (the best single individual set of descriptors) and consensus models for decomposition and MPs are publicly available on the OCHEM web site. The patent-mined data from this study are publicly downloadable from the same web site as well as available from FigShare [70] under a CC-BY license [71]. Users of the data are however strongly encouraged to cite this article as well as the data utilized as this work describes the details of the extraction process and data cleaning specifically.

## Discussion and conclusion

We have collected from the literature a large number of MPs and decomposition points of compounds. A number of technical challenges were solved to curate the data and transform the information from the text to computer readable formats. Many of these challenges were related to the ambiguous representation of information within chemical PATENTS.

As the abstraction used for text mining only requires a list of headings and paragraphs, application of the same methodology to other structured text such as journal articles and other properties would be a straightforward extension.

We have shown that models based on the data collected from the PATENTS provided similar or higher prediction ability, compared to the results from our previous study. This indicates the high quality of patent-mined data, which is similar to that of manually curated data from the literature.

The PATENTS data contained about 5.5 % of compounds, which decompose during melting. The separation of data into subsets of compounds, which decompose and do not decompose during melting increased the accuracy of the individual models for both properties. The use of SetCompare tools allowed for the identification of chemical features, which are important for the pyrolysis of chemical compounds. Moreover, a classification model, which can predict whether a compound will decompose during MP measurement, was also developed.

In our previous study [11] we suggested that the 691 outlying molecules could be enriched with decomposing structures. The classification model predicted 28 % of these molecules as decomposing while only 21 % were predicted for the rest of the COMBINED set. Thus, indeed, the outlying structures contained a significantly higher percentage of decomposing compounds. The model also predicted 22 and 14 % decomposing compounds for the set of 4.7k outliers (identified with *p* = 0.01, see Table 5) and the remaining compounds of the PATENTS set, respectively. The outlying compounds were therefore again enriched with decomposing compounds. This result suggests that the PATENTS dataset may still contain decomposing compounds, which were not annotated in the PATENTS literature. The presence of decomposing compounds in the training set of the non-decomposing subset for the development of the pyrolysis classification model could decrease its accuracy. The difference between the average numbers of predicted compounds for COMBINED and PATENTS sets was about 6 %, that is the percentage of decomposing compounds annotated in the PATENTS literature. Thus, the COMBINED set has about the same percentage of decomposing compounds as the PATENTS set. The pyrolysis compounds were excluded for development of the MP model but for better comparison with the previous model the analysis in Table 3 was performed without separation of both classes.

**Table 5:** RMSE of models developed with filtering of outliers

	No filtering	0.001 (N = 1414)^a^	0.01 (N = 4727)	0.1 (N = 21,928)
PATENTS				
CDK	38.9 (36.2)	38.9 (36.1)	38.8 (36.1)	38.9 (36.1)
Isida Fragmentor	38.5 (35.5)	38.4 (35.4)	38.3 (35.2)	38.2 (35.2)
ChemAxon	40.1 (37.1)	40 (37.1)	40.1 (37.1)	40.1 (37.2)
QNPR	39.7 (36.6)	39.7 (36.3)	39.4 (36)	39.2 (35.9)
E-state	38.3 (35.6)	38.1 (35.6)	38.1 (35.5)	38.0 (35.5)
Consensus	36.3 (33.3)	36.2 (33.3)	36.3 (33.2)	36.4 (33.5)
COMBINED				
CDK	51.6 (35.6)	51.3 (35.5)	50.8 (35.5)	49.9 (35.4)
Isida Fragmentor	47.6 (35.9)	47.5 (35.6)	47.2 (35.3)	47.3 (35.4)
ChemAxon	49.7 (36.5)	49.6 (36.5)	49.5 (36.4)	49 (36.5)
QNPR	50.2 (38.1)	50.5 (37.8)	49.9 (37.7)	49.5 (37.6)
E-state	45.9 (35.4)	46.1 (35.4)	45.8 (35.3)	45.8 (35.2)
Consensus	46.5 (33.4)	46.3 (33.4)	46.2 (33.3)	46.1 (33.4)

^a^The numbers in parentheses indicate the number of molecules detected as outliers and filtered from the PATENTS set. The RMSE values for the PATENTS set are calculated for all molecules in this set (including the outliers)

Using the repeated measurements in PATENTS we estimated the experimental error of MP measurements as σ = 35 °C for the PATENTS set. We showed that the estimated accuracy varied as a function of temperature and achieved the lowest error of σ = 32 °C for the drug-like region of the dataset. Problems such as difficulties with experimental measurements for high temperatures, errors with reporting these values (i.e. using threshold or typing errors), as well as polymorphism and the purity of analyzed chemical compounds likely contribute to the measurement error. The final consensus model achieved a precision, which was similar to the estimated experimental accuracy. Thus contrary to previous studies, which indicated that the accuracy of models for physicochemical properties is limited by the insufficient descriptors [72, 73], we can conclude that our results were rather limited by the experimental data accuracy.

A comparison of MLRA and SVM results developed using exactly the same sets of descriptors indicated significantly higher accuracy of the SVM models. This result suggests high non-linearity and interactions of descriptors, which is better modeled by the SVM method.

Because of the limitation on the computational resources, the grid search to select SVM parameters was done using only one set of descriptors, EFG, which contained the smallest number of non zero values. Even these calculations required about 15,000 core-hours. It is possible that selection of SVM parameters for each set could contribute better models. Considering that the grid search does not always provide the optimal set of parameters [74], more sophisticated algorithms based on evolutionary programming can be used thus contributing even more accurate models. Such study, however, is beyond the scope of this article.

The final consensus model developed in this study provided the best published prediction accuracy for the Bergström subset, RMSE = 31 °C, which is a 3 °C improvement in the result from our previous study (see Table 6) [11] and this corresponds to an almost 15 °C improvement of results from the original study [17] and other earlier studies using this set [15, 73].

**Table 6:** RMSE of the final consensus models developed in this and in the previous study [29]

Method	PATENTS set	Bergström	Bradley	OCHEM	Enamine	COMBINED
PATENTS + COMBINED	36.5 ± 0.1 (33.7)	31 ± 1 (29)	32.2 ± 0.6 (32.2)	37.9 ± 0.3 (33)	36.3 ± 0.3 (31.1)	36.8 ± 0.3 (32)
COMBINED	44.6 ± 0.1 (40.9)^a^	34 ± 1 (31)	32.6 ± 0.6 (33.1)	38 ± 0.3 (33.7)	36.8 ± 0.3 (31.5)	37.1 ± 0.3 (32.6)

^a^The results of the prediction of the PATENTS set using the model developed in our previous study [29]

Further progress in the prediction of MPs can be advanced by improvement in the accuracy of experimental measurements, as well as prediction of MP for different polymorphic and amorphic forms. This work, however, is unlikely to happen in the near future since it will require rather different approaches to the collection and handling of experimental MP data.

The prediction of MP itself has limited practical value. The main interest in this property is because of its possible use for the estimation of the solubility of chemical compounds using the general solubility equation (GSE) [19]

3 $$\log\,{\text{S}} = 0.5 - 0.01({\text{MP-25}}) - \log{\text{P}}$$

where logS is the intrinsic molar solubility and logP is the octanol/water partition coefficient. According to this equation, the prediction of MP with RMSE of 30 °C contributes 0.3 log unit to the error of the solubility prediction. The estimation of logS with an error of <0.5 log units is on the level of the experimental measurement accuracy [75] and thus is very valuable for the pharma industry. Unfortunately, as indicated by the recent benchmarking study of 18 approaches contributed by leading academic groups and chemical software providers [76], the estimation of logP is more challenging and can contribute about one log unit error. This can limit the application of GSE to new chemicals. However, if the applicability domain [59] of models is carefully addressed and extended with new measurements, the accuracy of logP predictions could be as low as 0.35 logP units for about 60 % out of 96k analyzed compounds [77]. Such an approach could enable a widespread use of the GSE equation to estimate the solubility of chemical compounds.

As an illustrative example we applied the GSE to predict logS for N = 1311 molecules from our previous study [78]. The logP values were obtained using ALOGPS 2.1 program [79], which is also available as part of the OCHEM descriptors. Equation (3) gave a calculated RMSE of 0.84 ± 0.02. The same accuracy was calculated notwithstanding whether the consensus or model based on the E-state descriptors was used. While this error was higher than the RMSE of 0.62 calculated for the data in the original study the results obtained in this study did not use any information about the target property. The GSE water solubility model, which is based on E-state indices and thus requires lower computational resources, was made publicly available on the OCHEM web site.

The development and public availability of computational models developed with an increasing volume of publicly available data mined from the published literature is important to the development of better QSAR/QSPR models and their wider acceptance by academia, industry and chemical authorities [80].

## Additional files

10.1186/s13221-016-0113-2 Protocols used to develop the melting point consensus model.
10.1186/s13221-016-0113-2 RMSE of LibSVM models calculated with different sets of descriptors.

## Abbreviations

ADMET: absorption, distribution, metabolism, exertion and toxicity
ASNN: associative neural network
CV: cross-validation
logP: octanol/water partition coefficient
logS: water solubility
MLRA: multiple linear regression analysis
MP: melting point
OCHEM: on-line chemical database and modeling environment, http://ochem.eu
QSAR: quantitative structure activity relationship
QSPR: quantitative structure property relationship
RMSE: root mean squared error
SGML: standard generalized markup language
SVM: support vector machine
USPTO: United States Patent and Trademark Office
XML: extensible markup language
