# Supplementary material for: The development of models to predict melting and pyrolysis point data associated with several hundred thousand compounds mined from PATENTS
Source: J Cheminform. 2016 Jan 22;8:2. doi: 10.1186/s13321-016-0113-y (PMC4724158; doi:10.1186/s13321-016-0113-y)
Supplement: Supplementary file 1 — 10.1186/s13221-016-0113-2 Protocols used to develop the melting point consensus model. [file 13321_2016_113_MOESM1_ESM.pdf]

Supplementary Information to article:

The development of models to predict melting and pyrolysis point data associated with several hundreds thousands compounds mined from patents

Igor V. Tetko,[1,*] Daniel Lowe[2] and Antony J. Williams[3]

1) Helmholtz Zentrum München für Gesundheit und Umwelt (HMGU), Institute of Structural Biology, Ingolstaedter Landstrasse 1, b. 60w, D-85764 Neuherberg, Germany and BigChem GmbH, D-85764 Neuherberg, Germany i.tetko@helmholtz-muenchen.de
2) NextMove Software Limited, Innovation Centre (Unit 23), Cambridge Science Park, Cambridge CB4 0EY, UK, daniel@nextmovesoftware.com
3) ChemConnector Inc., 904 Tamaras Circle, Wake Forest, NC-27587, USA, Williams.Antony@epa.gov

Description of the protocols used to develop the Melting Point consensus model.

Overview 2
Step 1. Data upload 2
Step 2. Identification of LibSVM parameters. 3
Step 2.1 Starting model development. 3
2.2 Selection of the dataset, method and validation protocol to identify the best set of LibSVM parameters. 4
2.3 Selection of preprocessing options 5
2.4 Selection of Extended Functional Groups (EFGs) as descriptors. 6
2.5 The descriptor unsupervised filtering options 7
2.6 Grid search to detect optimized parameters. 8
2.7 The calculations are launched. 9
2.8 Selection of Pending task window to monitor the execution of tasks. 10
2.9 Monitoring of the calculations in the PendingTask browser. 10
2.10 The model is calculated. 11
2.11 The model is open (green button on the previous plot) and saved. 11
2.12 The optimal parameters of the model are used to create a new method template. 12
3. The development of multiple models is started using model template with optimized LibSVM parameters. 13
3.1 Consensus model developed using the "Simple average" option. 14
Step 4. The calculated models are accessed in the table – like Comprehensive Modeling (CM) view from "Baskets" web page. 15
Step 4.1 An overview of the developed models in the Comprehensive Modeling (CM) window. 15

Overview

Below, we summarize steps used for the development of the consensus model. The illustrative Figures are followed by a brief description of the respective step.

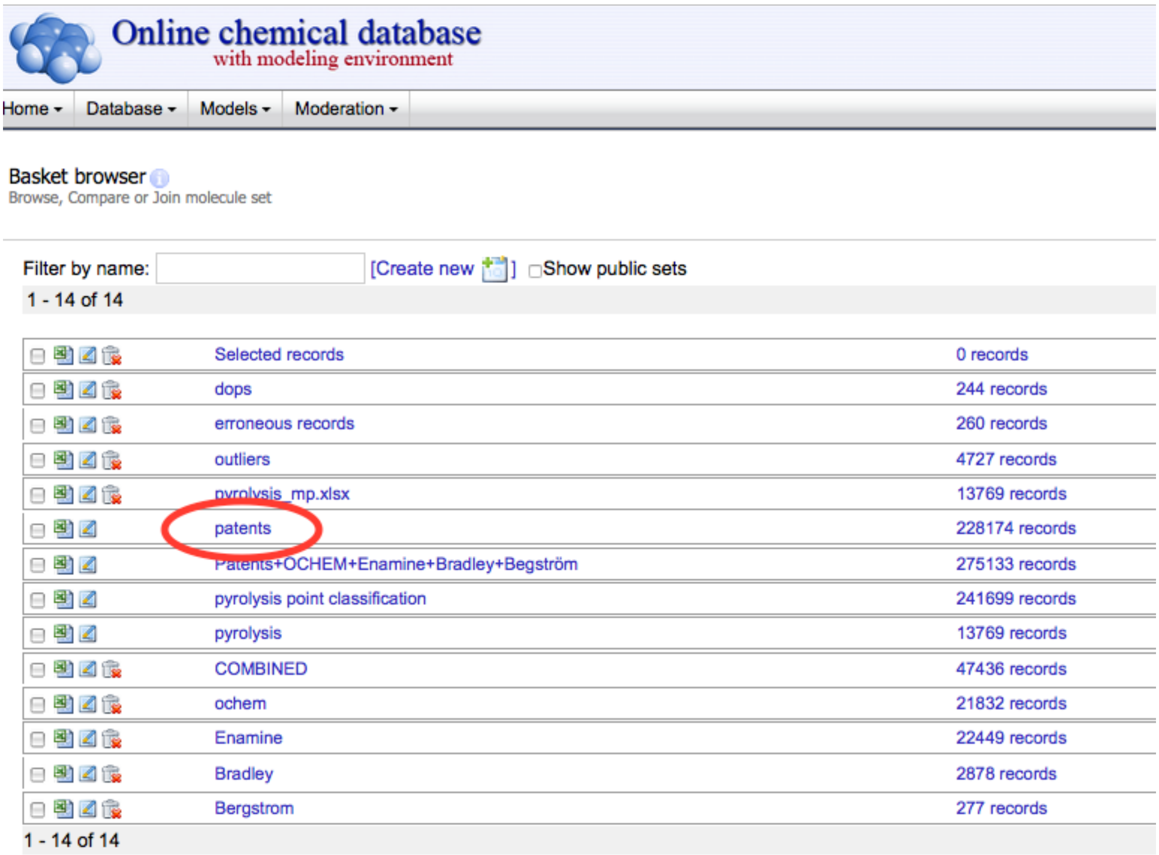

Step 1. Data upload.

The data for the Melting Point (MP) model are uploaded in the OCHEM database (for these steps see tutorial at https://www.youtube.com/watch?v=vdJWiS4wSaQ) and formed the basket "patents".

Step 2. Identification of LibSVM parameters.

A preliminary analysis using a grid search (see Methods) was run to select the optimal parameters for the SVM model. It included the following several substeps.

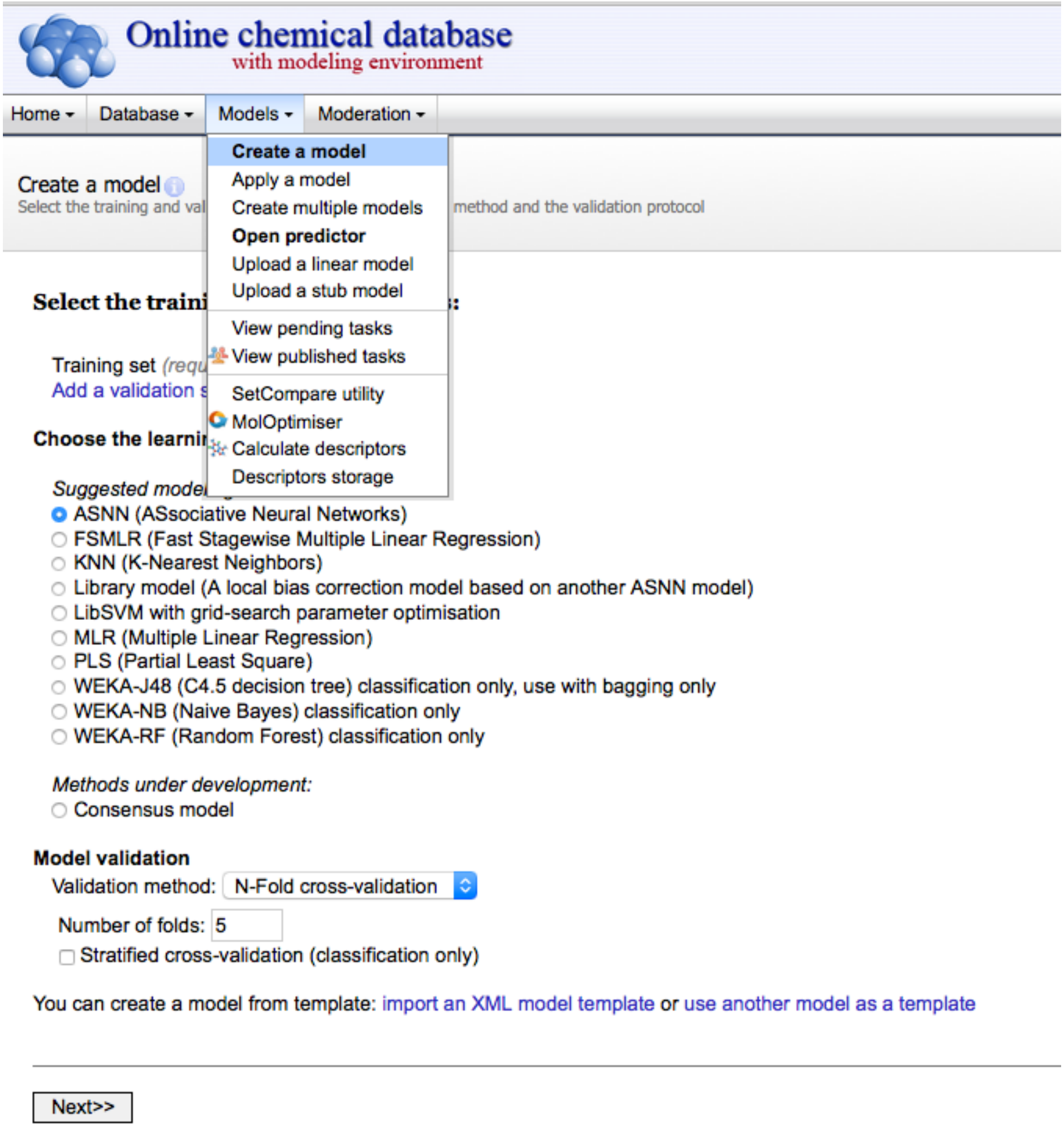

Step 2.1 Starting model development.

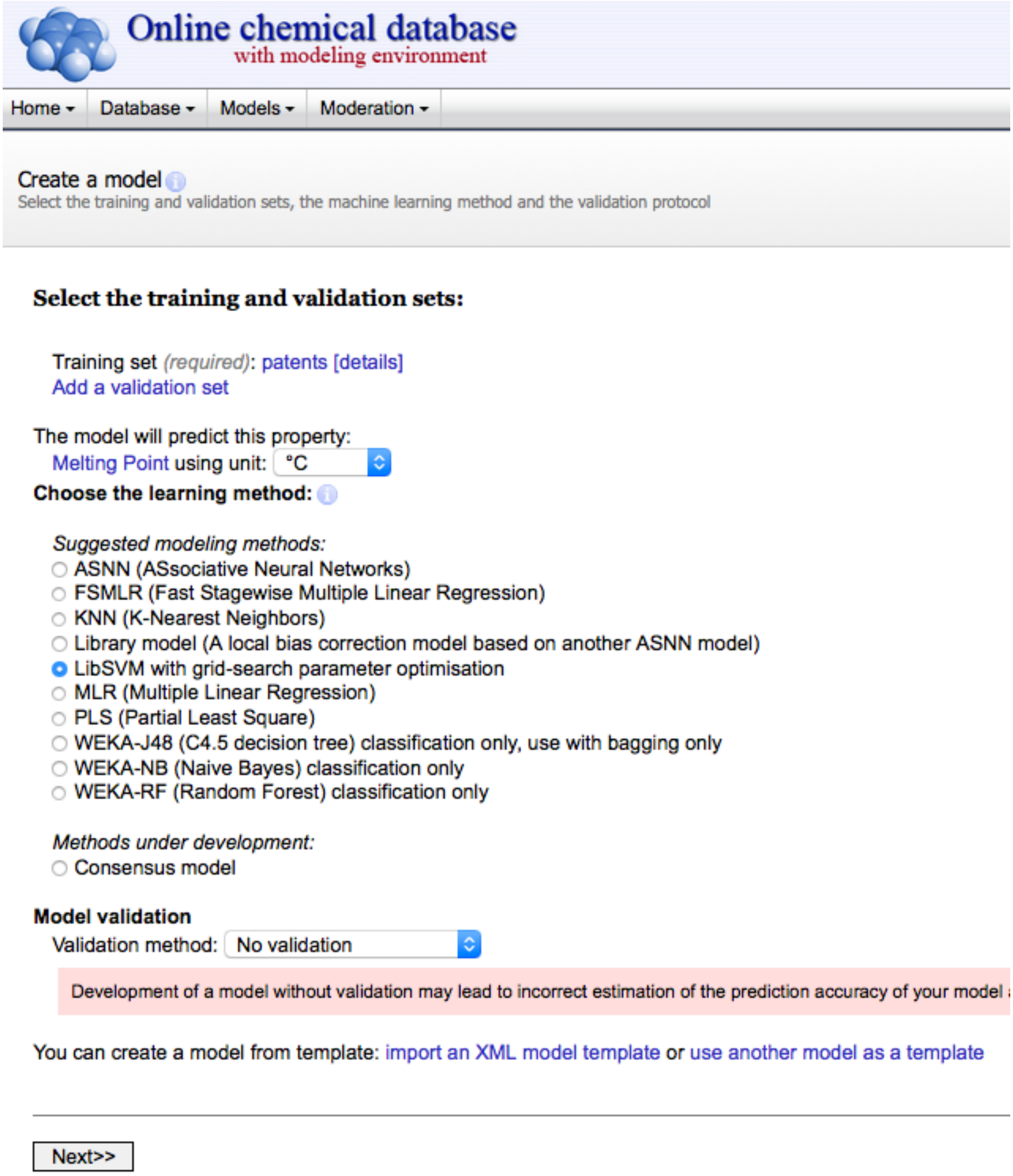

2.2 Selection of the dataset, method and validation protocol to identify the best set of LibSVM parameters.

Since the calculations in this step required very large CPU resources and LibSVM parameters were optimized for the whole set, "no validation" method was used. This method is normally not recommended since it can result in data overfitting. In this case only three LibSVM parameters were optimized for N = 228k compounds and thus there was no problem with overfitting. Notice that these calculations required more than 600 CPU-days of calculations.

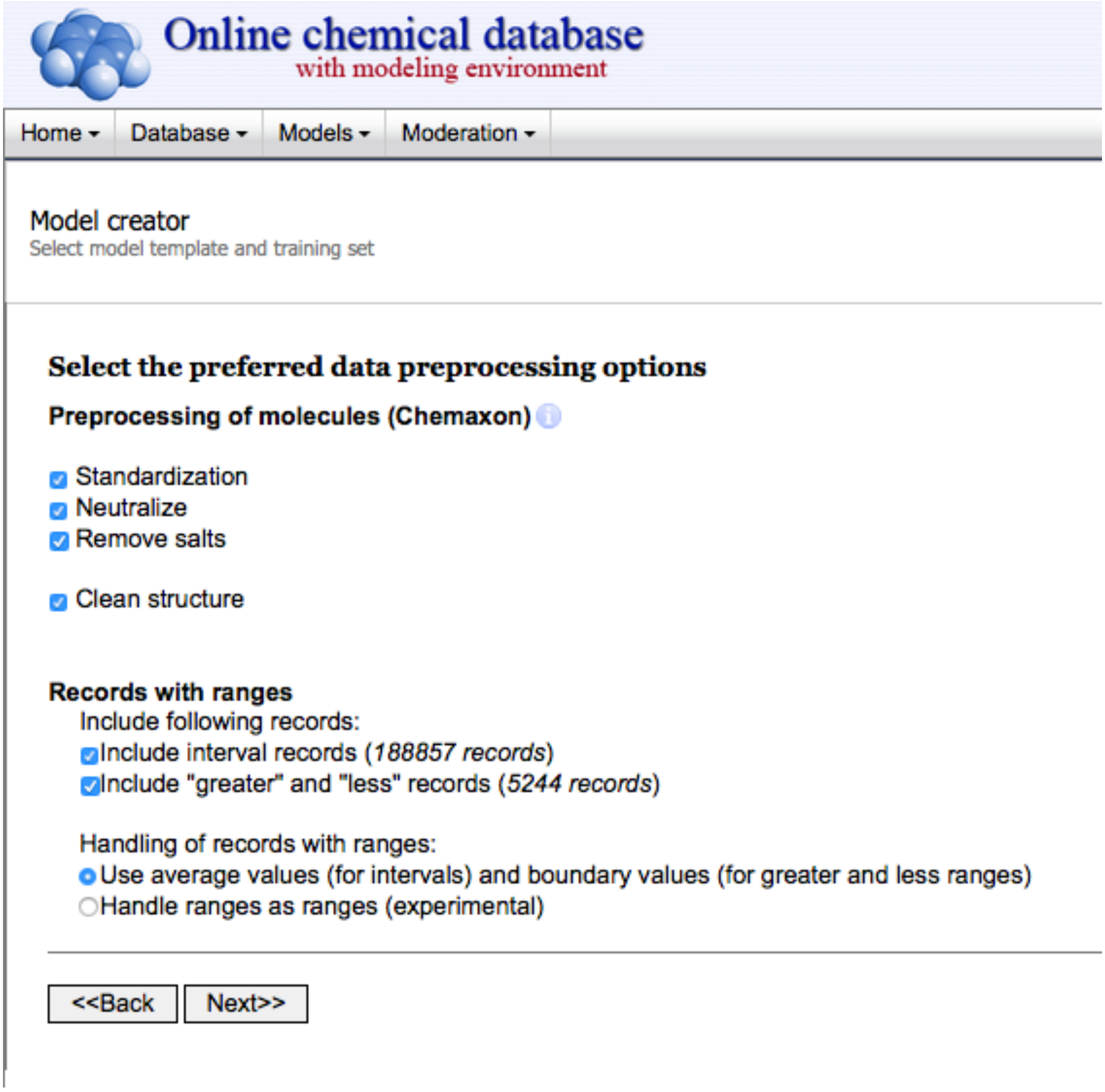

2.3 Selection of preprocessing options.

The options included use of records with intervals and ranges for model development.

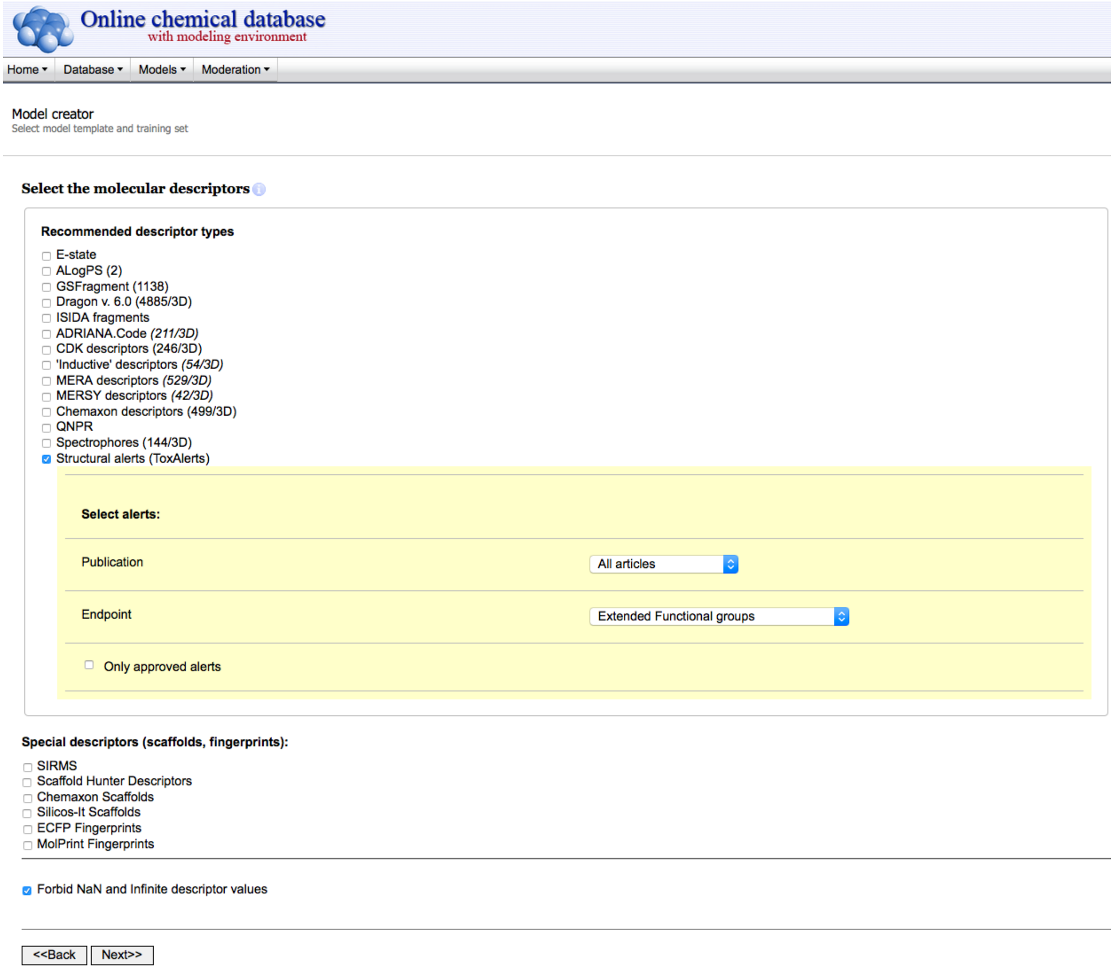

2.4 Selection of Extended Functional Groups (EFGs) as descriptors.

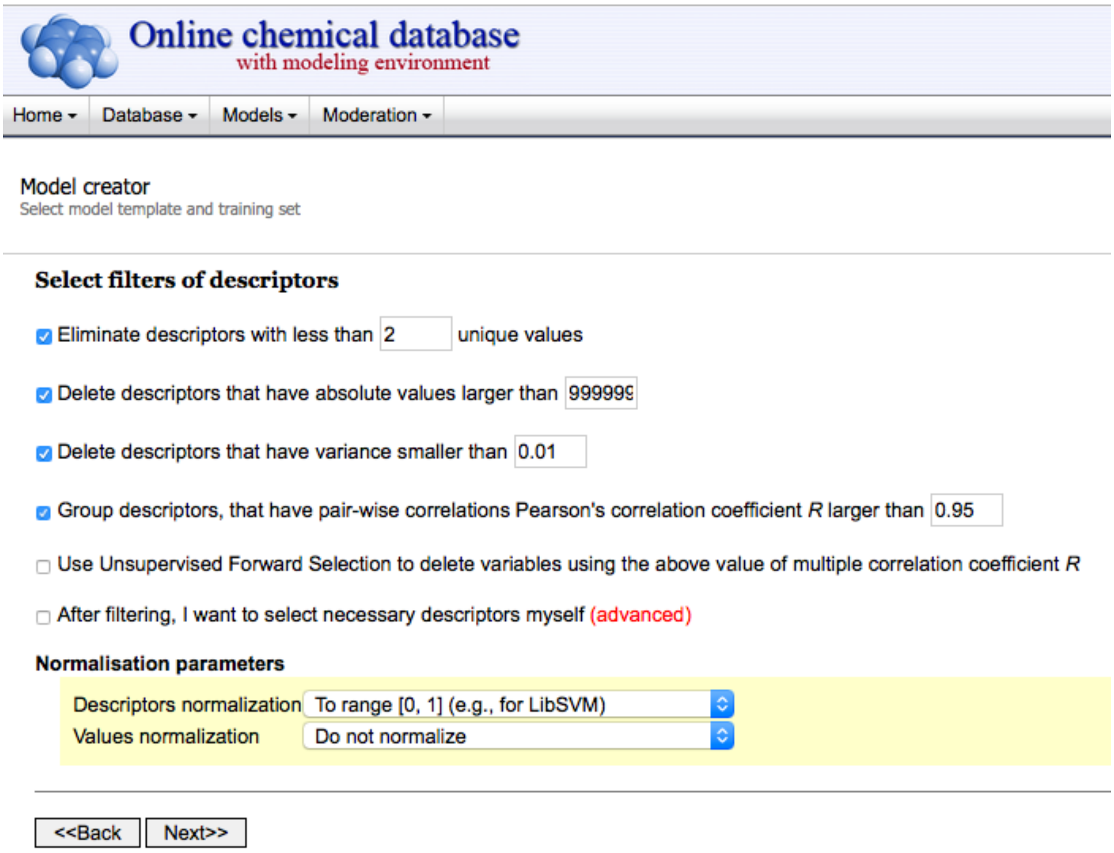

2.5 The descriptor unsupervised filtering options

The normalization to [0,1] interval was selected. Other pre-processing options were used as default values.

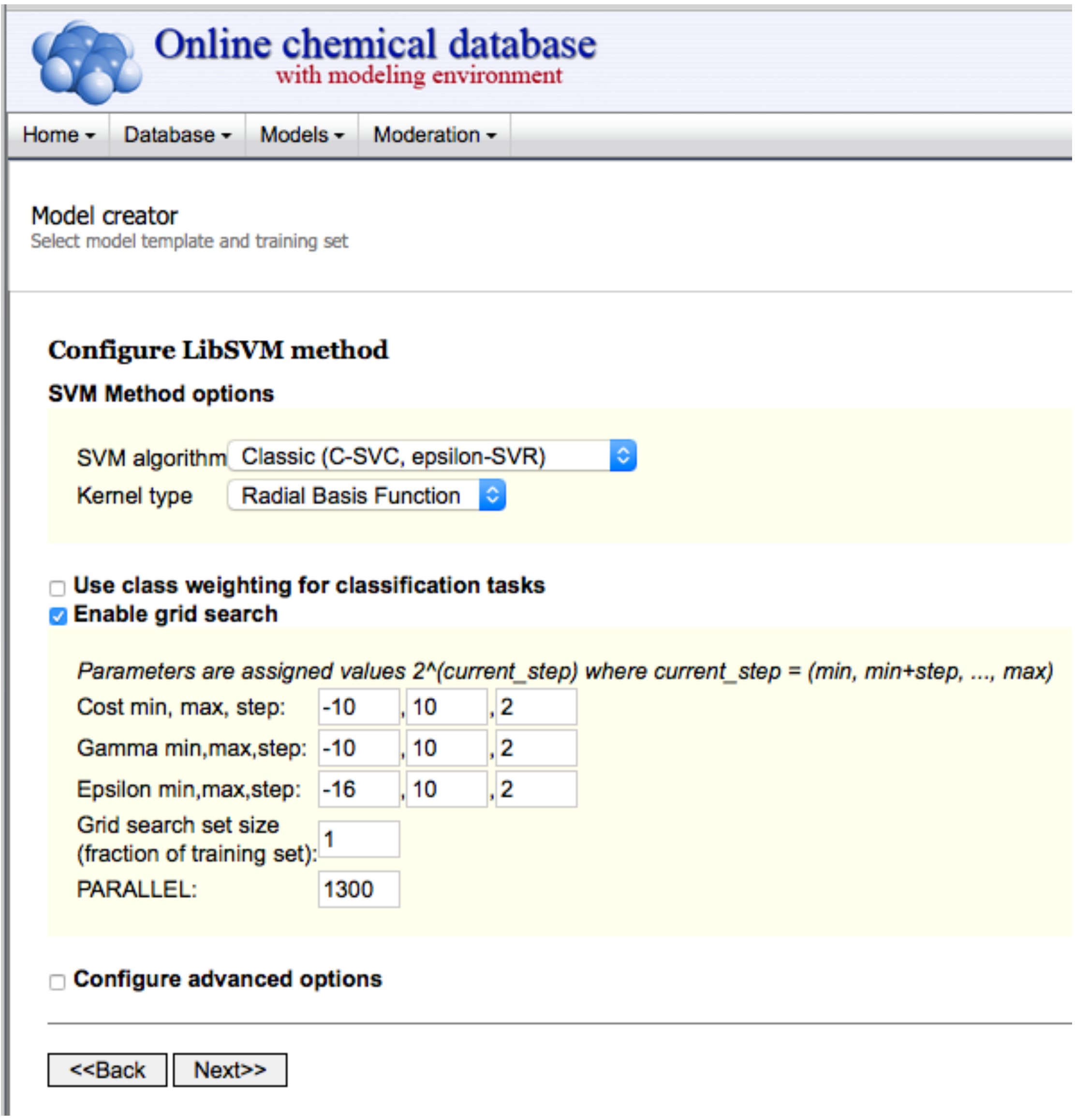

2.6 Grid search to detect optimized parameters.

The calculations were configured to run in parallel on 1300 servers (option PARALLEL) and using the whole training set (fraction = 1).

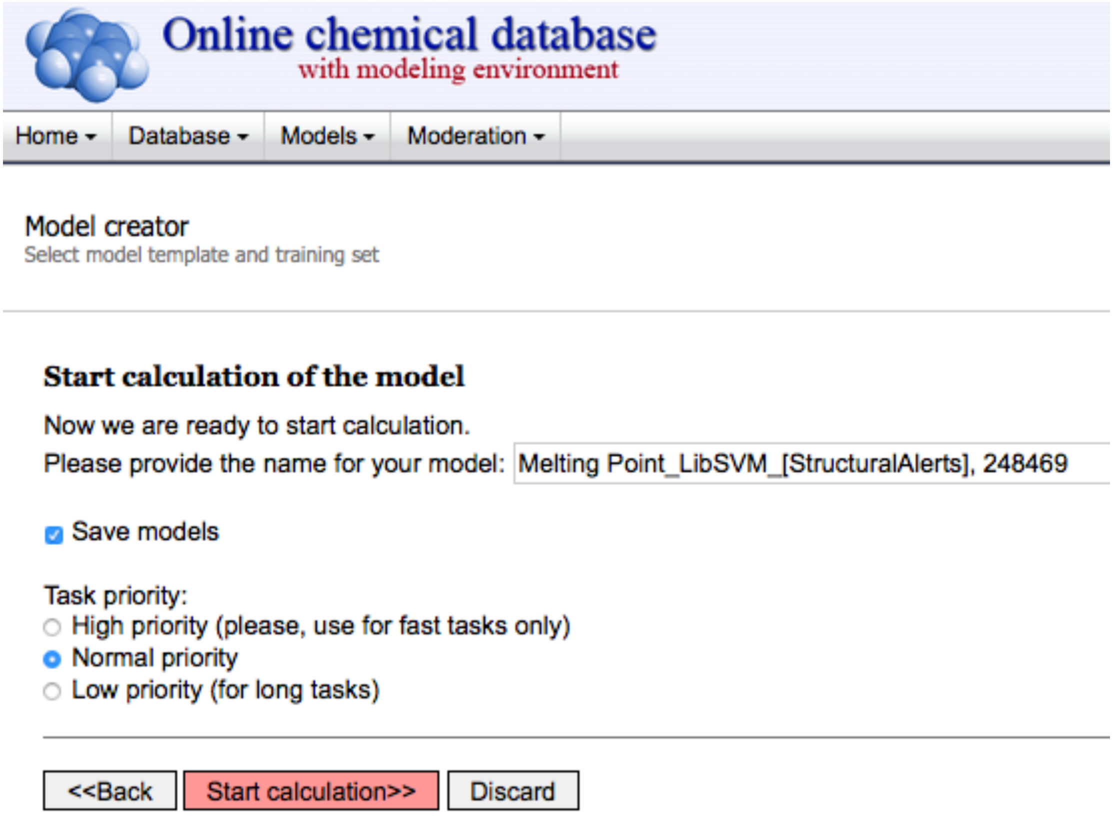

2.7 The calculations are launched.

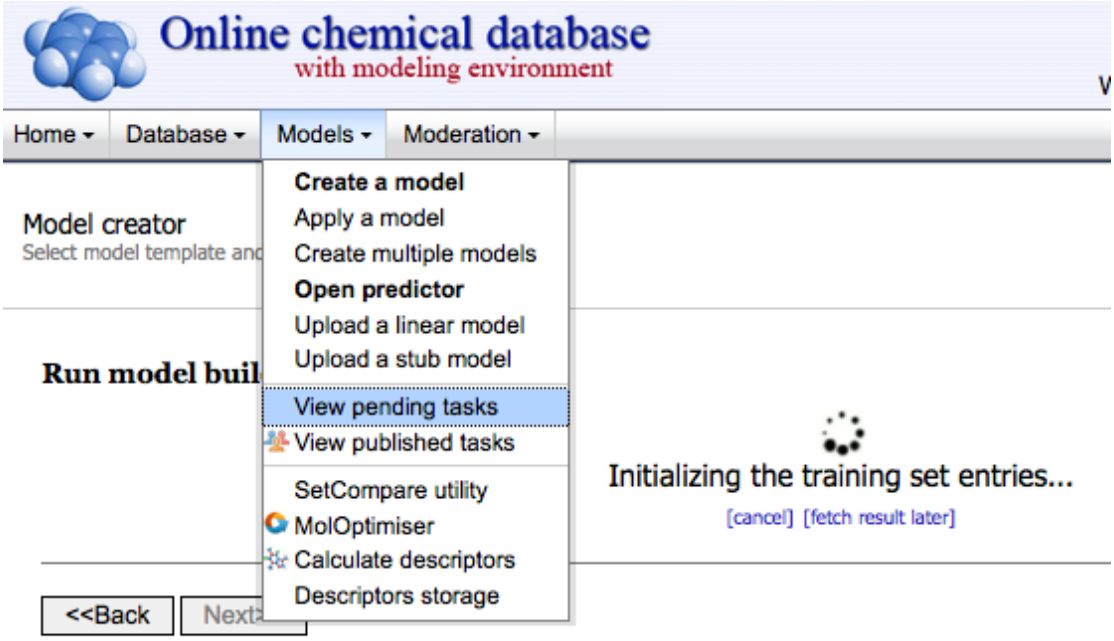

2.8 Selection of Pending task window to monitor the execution of tasks.

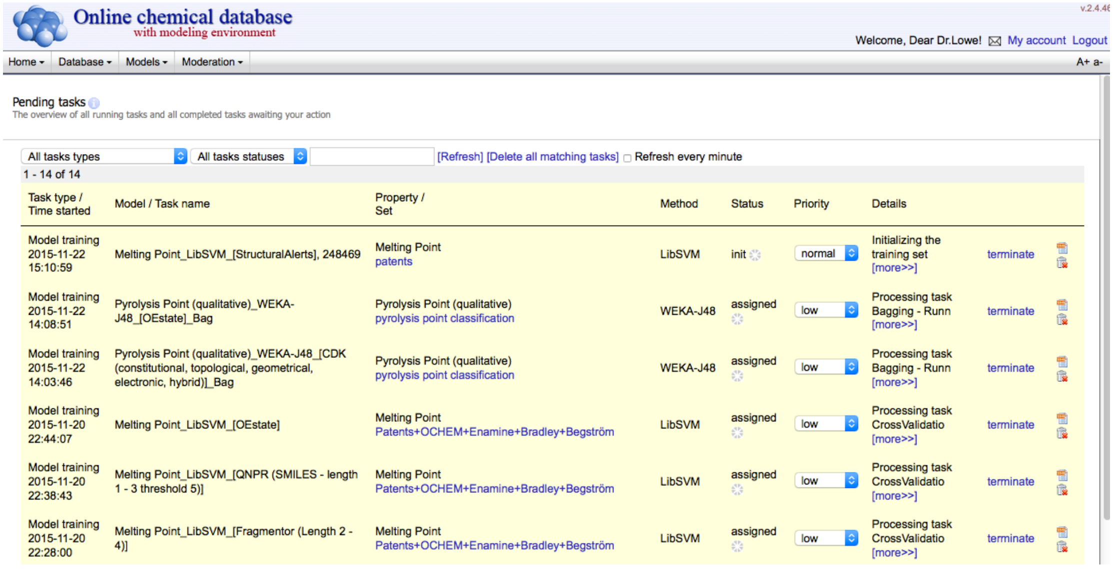

2.9 Monitoring of the calculations in the PendingTask browser.

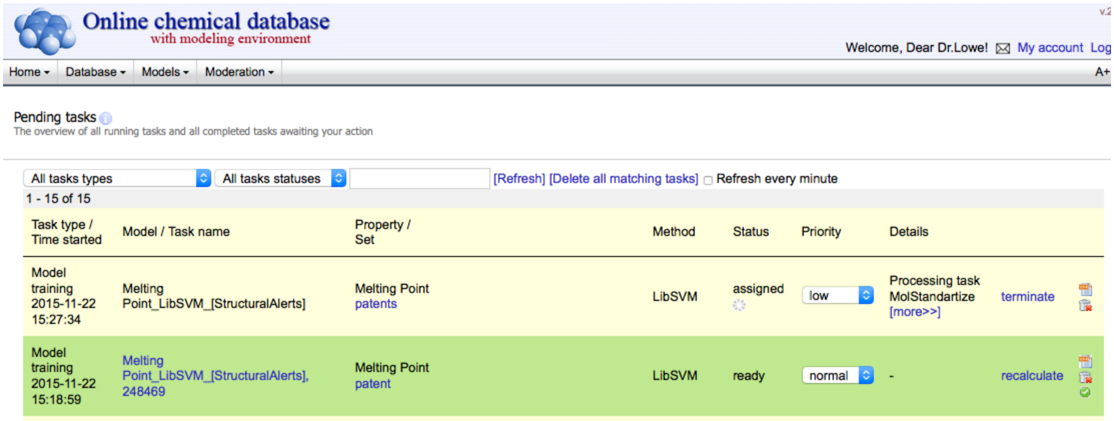

2.10 The model is calculated.

The optimal parameters for the training set were calculated. They could be exported using XML icon at the left side of model row.

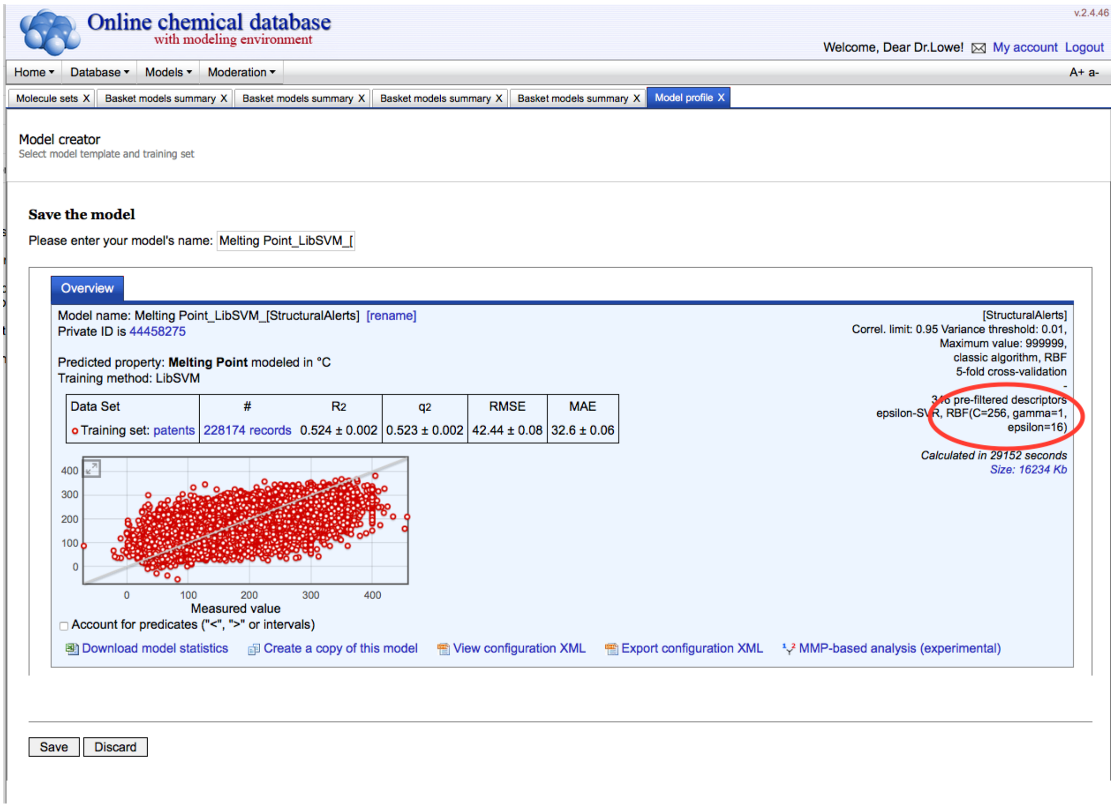

2.11 The model is open (green button on the previous plot) and saved.

The optimized parameters for LibSVM are shown. They are part of the model template, which can be exported using the "Export configuration XML" button. The exported template can be used to develop new models (see step 2.1 – "import an XML model template") using the same setting.

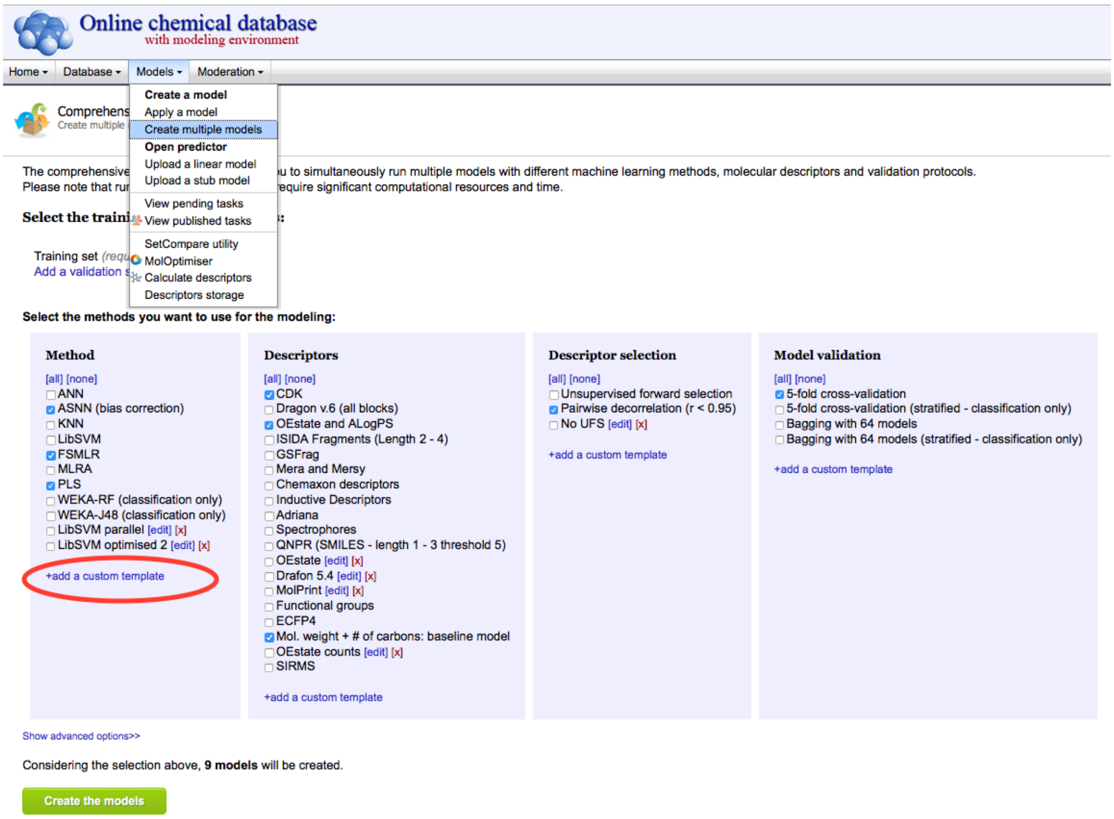

2.12 The optimal parameters of the model are used to create a new method template.

The “Comprehensive Modeling” mode was opened and a template was created using the “add a custom template on “Models/Create multiple models” menu. The previously selected model with optimized parameters was used as the template. An experienced user can also manually edit the XML file in the browser. In this example template with optimized LibSVM parameters was created. In a similar way new templates for descriptors, unsupervised descriptor selection and model validation can be created. They could be combined to create multiple models.

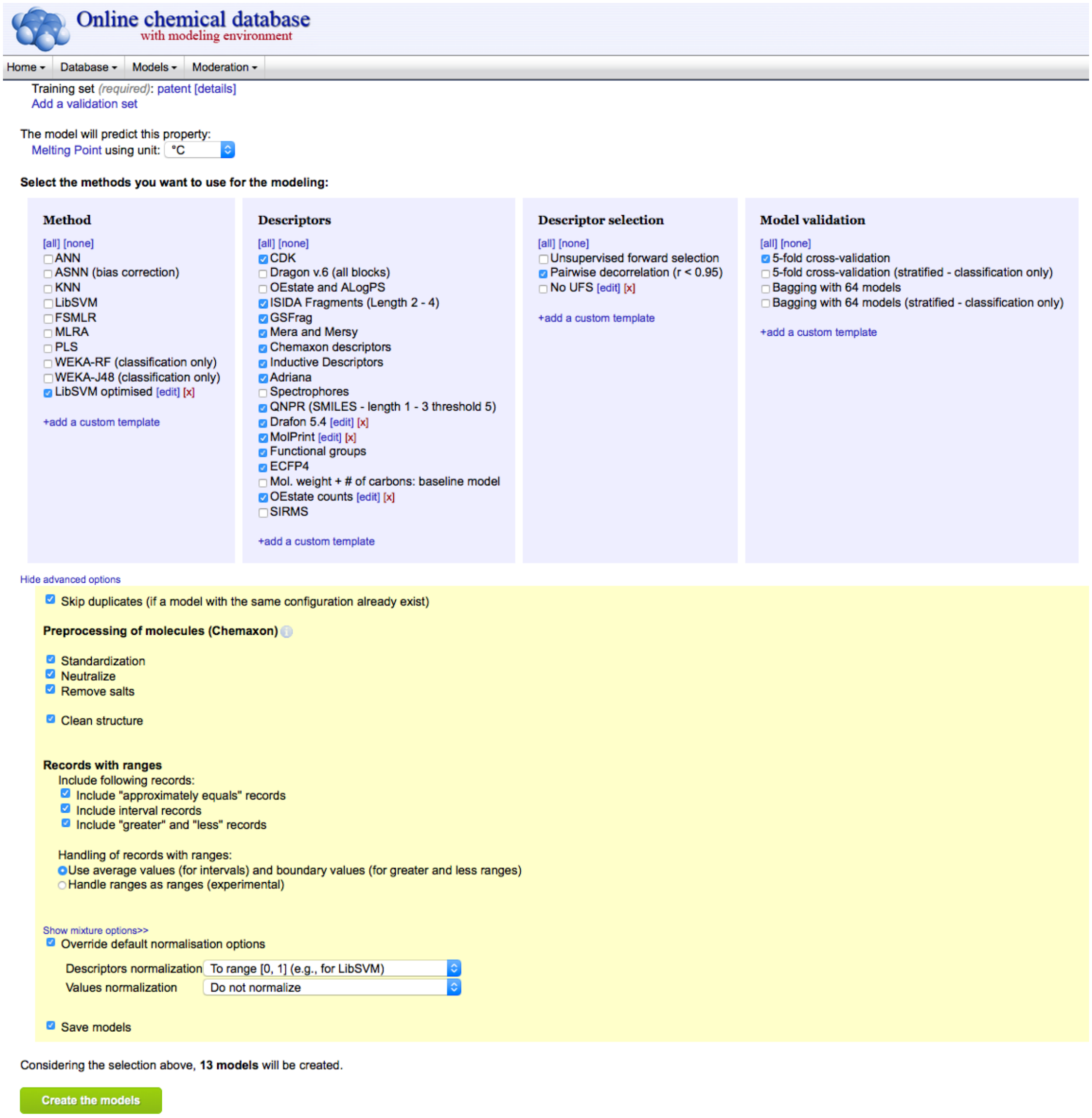

3. The development of multiple models is started using model template with optimized LibSVM parameters.

For this article we used default settings for descriptors, descriptor selection and validation protocols as shown in the Figure above.

Models calculation was monitored in the Pending Task menu (see 2.8 and 2.9). Once models were finished, they were stored (each model individually, see step 2.11). After that they were used to develop a Consensus model using the "Consensus model" template and the same steps (2.1 and 2.2) used for the LibSVM model.

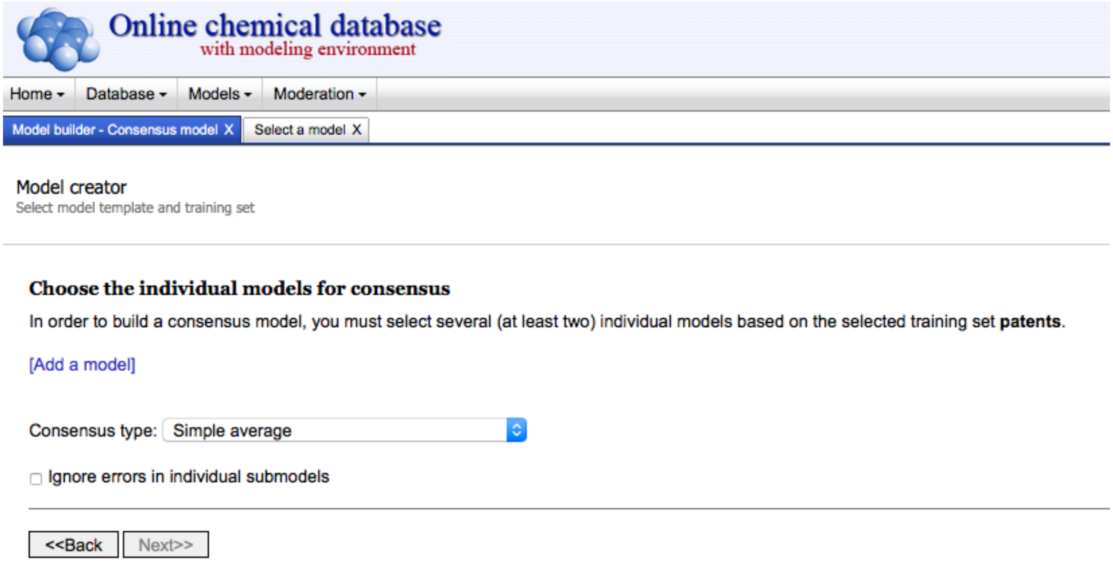

3.1 Consensus model developed using the "Simple average" option.

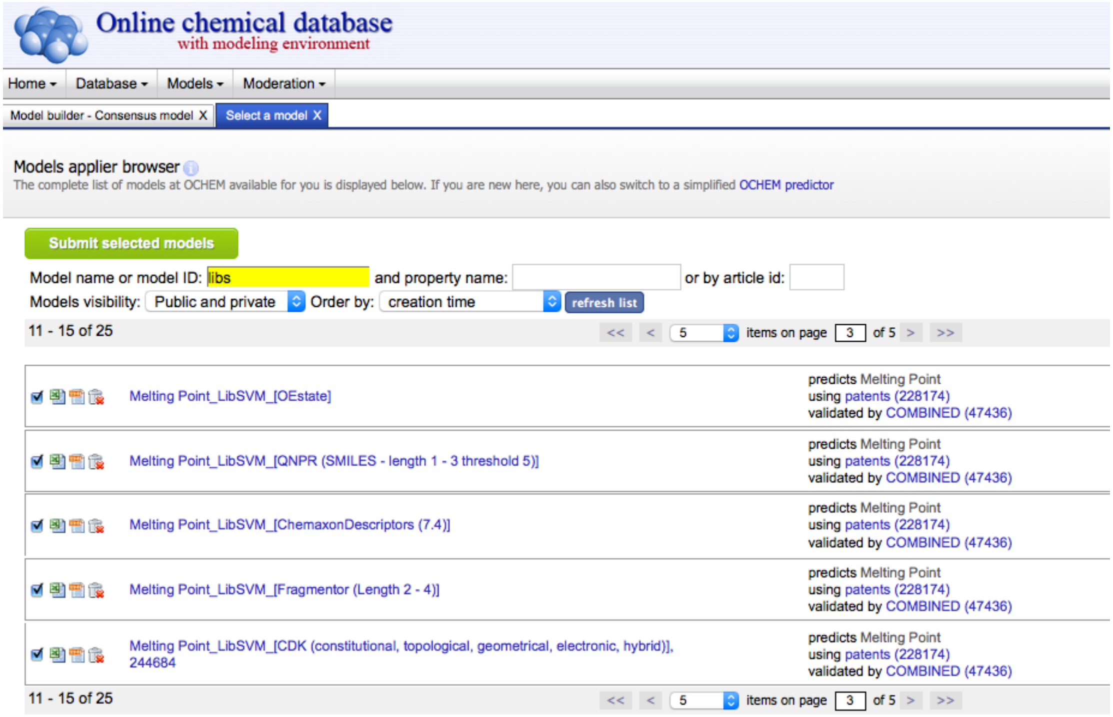

3.2 Selection of saved models for averaging in "Consensus model".

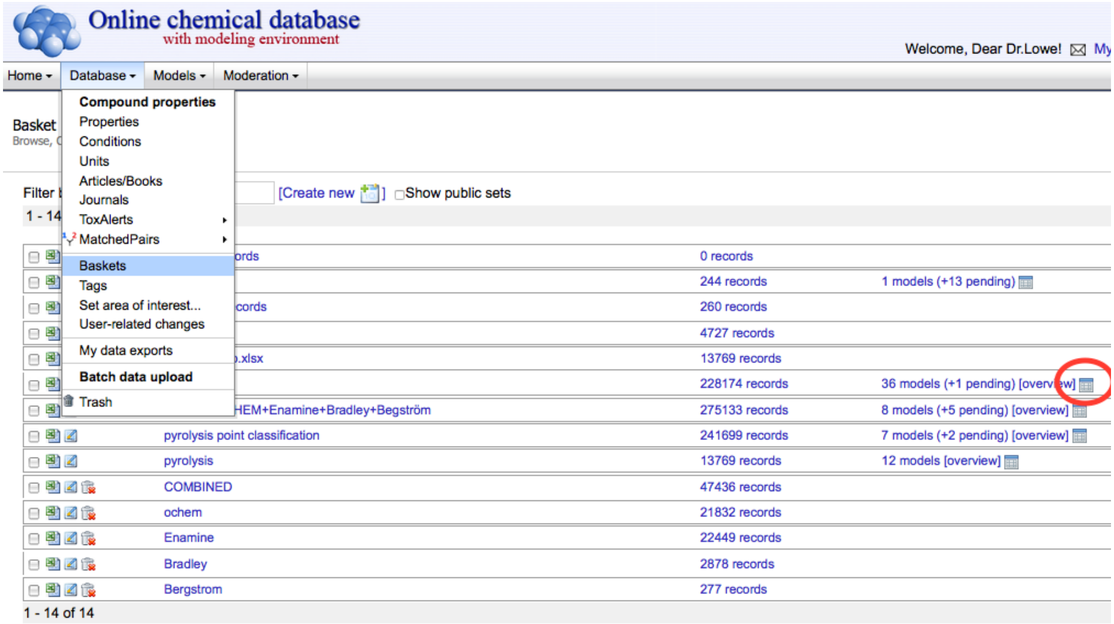

Step 4. The calculated models are accessed in the table – like Comprehensive Modeling (CM) view from "Baskets" web page.

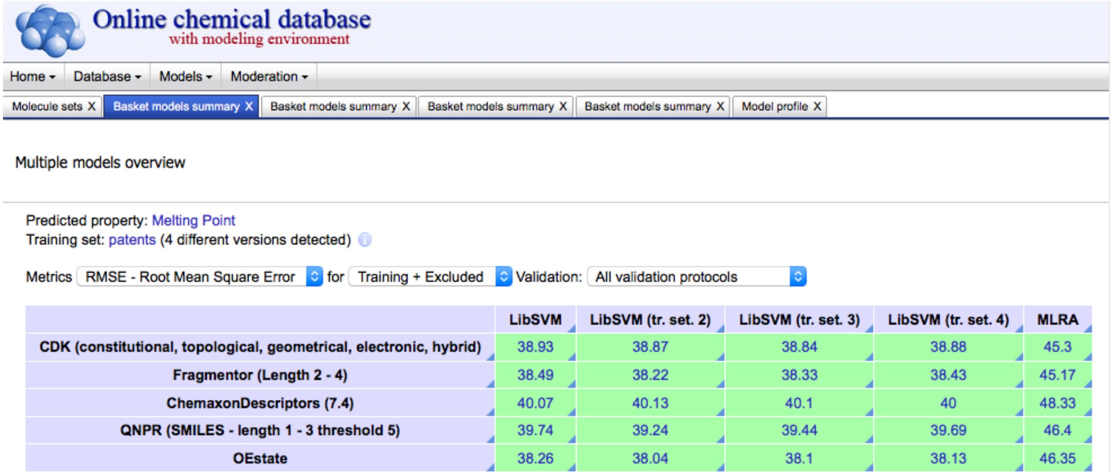

	LibSVM	LibSVM (tr. set. 2)	LibSVM (tr. set. 3)	LibSVM (tr. set. 4)	MLRA
CDK (constitutional, topological, geometrical, electronic, hybrid)	38.93	38.87	38.84	38.88	45.3
Fragmentor (Length 2 - 4)	38.49	38.22	38.33	38.43	45.17
ChemaxonDescriptors (7.4)	40.07	40.13	40.1	40	48.33
QNPR (SMILES - length 1 - 3 threshold 5)	39.74	39.24	39.44	39.69	46.4
OEstate	38.26	38.04	38.1	38.13	46.35

Step 4.1 An overview of the developed models in the Comprehensive Modeling (CM) window.

Models developed with individual sets of descriptors are shown. The columns correspond to sets formed by the exclusion of outlying molecules with different p-values. The models developed using MLRA calculate with much lower accuracy.
