# Supplementary material for: The development of models to predict melting and pyrolysis point data associated with several hundred thousand compounds mined from PATENTS
Source: J Cheminform. 2016 Jan 22;8:2. doi: 10.1186/s13321-016-0113-y (PMC4724158; doi:10.1186/s13321-016-0113-y)
Supplement: Supplementary file 2 — 10.1186/s13221-016-0113-2 RMSE of LibSVM models calculated with different sets of descriptors. [file 13321_2016_113_MOESM2_ESM.docx]

Table S1. RMSE of LibSVM models calculated with different sets of descriptors

| Descriptors | RMSE | R2 | Q2 | MAE |
| --- | --- | --- | --- | --- |
| E-state count | 38.3 | 0.61 | 0.61 | 28.9 |
| ISIDA Fragmentor | 38.5 | 0.61 | 0.61 | 29 |
| CDK | 38.9 | 0.6 | 0.6 | 29.5 |
| QNPR | 39.7 | 0.58 | 0.58 | 30.2 |
| ChemAxon | 40.1 | 0.58 | 0.58 | 30.5 |
| GSFrag | 42.1 | 0.53 | 0.53 | 32.3 |
| Dragon | 42.4 | 0.53 | 0.52 | 32.8 |
| EFG | 42.5 | 0.52 | 0.52 | 32.6 |
| Mera, Mersy | 42.6 | 0.52 | 0.52 | 32.8 |
| Adriana | 42.9 | 0.51 | 0.51 | 32.9 |
| Inductive | 49.8 | 0.35 | 0.34 | 39.3 |
| MolPrint | 60.8 | 0.02 | 0.02 | 49.5 |
| ECFP4 | 61.3 | 0 | 0 | 50 |
